# An anti-inflammatory and anti-fibrotic proprietary Chinese medicine nasal spray designated as Allergic Rhinitis Nose Drops (ARND) with potential to prevent SARS-CoV-2 coronavirus infection by targeting RBD (Delta)- angiotensin converting enzyme 2 (ACE2) binding

**DOI:** 10.1186/s13020-022-00635-2

**Published:** 2022-07-27

**Authors:** Ka Man Yip, Kwan Ming Lee, Tzi Bun Ng, Shujun Xu, Ken Kin Lam Yung, Shaogang Qu, Allen Ka Loon Cheung, Stephen Cho Wing Sze

**Affiliations:** 1 Department of Biology, Faculty of Science, Hong Kong Baptist University, Kowloon Tong, Hong Kong, Special Administrative Region China; 2 Golden Meditech Center for NeuroRegeneration Sciences, Hong Kong Baptist University, Kowloon Tong, Hong Kong, Special Administrative Region China; 3 School of Biomedical Sciences, Faculty of Medicine, The Chinese University of Hong Kong, Shatin, Hong Kong, Special Administrative Region China; 4 Department of Neurology, Nanfang Hospital, Southern Medical University, Guangzhou, China; 5 Guangdong-Hong Kong-Macao Greater Bay Area Center for Brain Science and Brain-Inspired Intelligence, Guangzhou, 510515 Guangdong China; 6 Key Laboratory of Mental Health of the Ministry of Education, Southern Medical University, Guangzhou, 510515 Guangdong China

**Keywords:** ARND, COVID-19 Delta, Pseudovirus infection, Cytokine storm, Fibrosis

## Abstract

**Background:**

Since the outbreak of COVID-19 has resulted in over 313,000,000 confirmed cases of infection and over 5,500,000 deaths, substantial research work has been conducted to discover agents/ vaccines against COVID-19. Undesired adverse effects were observed in clinical practice and common vaccines do not protect the nasal tissue. An increasing volume of direct evidence based on clinical studies of traditional Chinese medicines (TCM) in the treatment of COVID-19 has been reported. However, the safe anti-inflammatory and anti-fibrotic proprietary Chinese medicines nasal spray, designated as Allergic Rhinitis Nose Drops (ARND), and its potential of re-purposing for suppressing viral infection via SARS-CoV-2 RBD (Delta)- angiotensin converting enzyme 2 (ACE2) binding have not been elucidated.

**Purpose:**

To characterize ARND as a potential SARS-CoV-2 entry inhibitor for its possible preventive application in anti-virus hygienic agent.

**Methods:**

Network pharmacology analysis of ARND was adopted to asacertain gene targets which were commonly affected by COVID-19. The inhibitory effect of ARND on viral infection was determined by an in vitro pseudovirus assay. Furthermore, ARND was confirmed to have a strong binding affinity with ACE2 and SARS-CoV-2 spike-RBD (Delta) by ELISA. Finally, inflammatory and fibrotic cell models were used in conjunction in this study.

**Results:**

The results suggested ARND not only inhibited pseudovirus infection and undermined the binding affinity between ACE2 and the Spike protein (Delta), but also attenuated the inflammatory response upon infection and may lead to a better prognosis with a lower risk of pulmonary fibrosis. The data in this study also provide a basis for further development of ARND as an antiviral hygienic product and further investigations on ARND in the live virus, in vivo and COVID-19 patients. ARND holds promise for use in the current COVID-19 outbreak as well as in future pandemics.

**Conclusion:**

ARND could be considered as a safe anti-SARS-CoV-2 agent with potential to prevent SARS-CoV-2 coronavirus infection**.**

**Graphical abstract:**

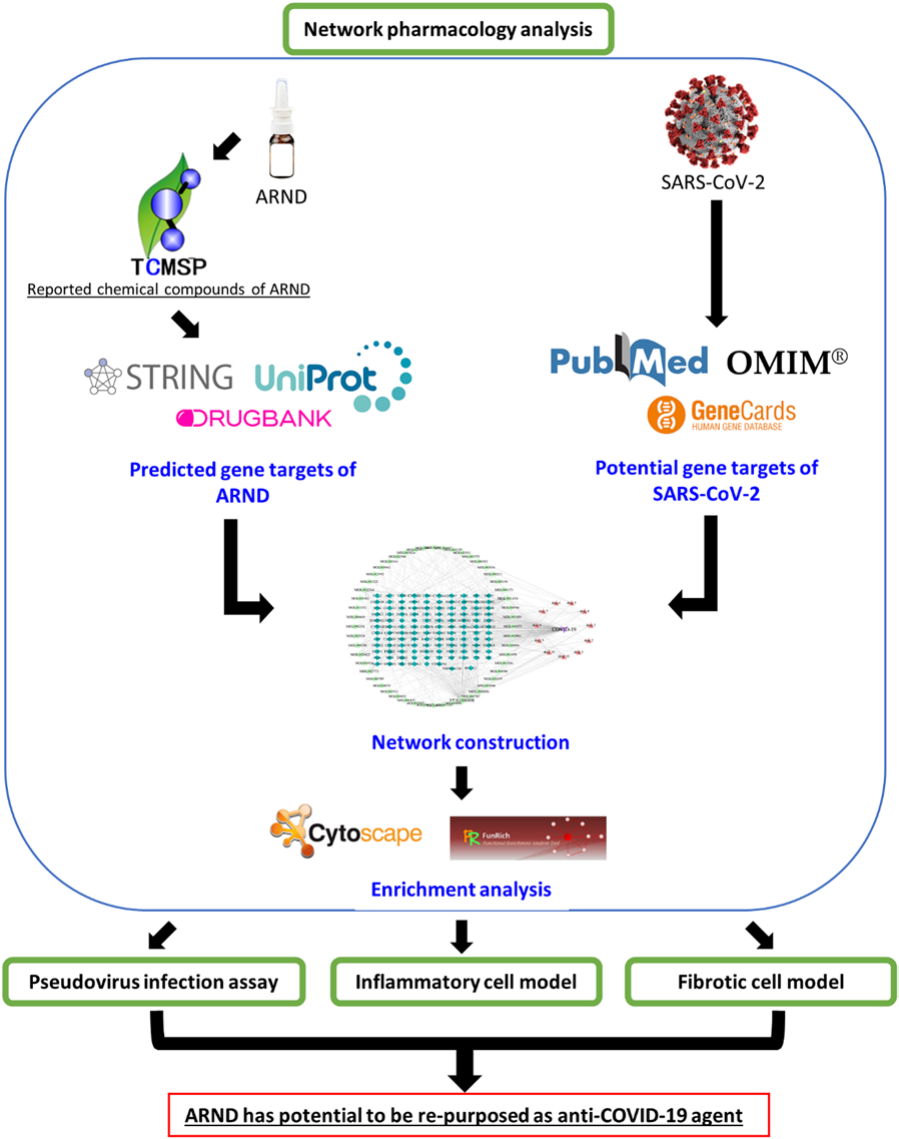

**Supplementary Information:**

The online version contains supplementary material available at 10.1186/s13020-022-00635-2.

## Introduction

Coronavirus Disease 2019 (COVID-19) is the disease caused by the virus designated as 2019-nCoV, which carries the scientific name of ‘severe acute respiratory syndrome coronavirus-2 (SARS-CoV-2) [1]. As of January 18, 2022, COVID-19 has speedily disseminated to many different countries and regions with 326,279,424 confirmed cases and 5,536,009 deaths all over the world according to statistics of the World Health Organization (WHO), having a major deleterious impact on humans and society, and posing an unprecedented, immense menace to the global public health system. SARS-CoV-2 is exceedingly contagious. Infants and children are generally vulnerable whereas the elderly and patients with chronic disease have a heightened risk of infection [2].

Inflammation is the major pathological response of the body after SARS-CoV-2 infection, in which elevated cytokine levels, such as IL-1β, IL-6, TNF-α, NOS2 and CCL2 are often observed in COVID-19 patients [3–6]. Excessive inflammation and thus ‘cytokine storm’ are frequently the outcomes. Cytokine storm is a process of enhanced monocyte recruitment and differentiation but it causes damage to the body’s own immune system at the same time. Nuclear factor-κB (NF-κB), including NF-κB2 (alternatively called p52) and c-Rel, is a member of a family of inducible transcription factors that controls a vast number of genes involved in immunological and inflammatory responses [7]. It could be activated by non-structural protein region open reading frame (ORF)-3a and 7a as well as structural protein membrane (M), and nucleocapsid (N) of SARS-CoV-2 [8]. Nitric oxide synthase 2 (NOS2) or inducible NOS, is also involved in the inflammatory responses that occur after infection or tissue injury [9]. Increased iNOS for NO production against the virus may lead to lung injury under the cytokine storm [10]. The abovementioned evidence indicates that suppression of these cytokines of the cytokine storm may help to curb the excessive inflammatory response after viral infection [11].

Lung fibrosis indicates a hallmark symptom of COVID-19 and a major complication to COVID-19 survivors. It is a consequence of severe lung injury that results from disordered wound healing process [12]. TGF-β is involved in a continuous immune reaction in severe COVID-19 patients [13] and its overexpression engenders serious fibrosis in the lungs [14]. A previous study proved that cells treated with TGF-β shifted more toward mesenchymal state in murine NSCLC cells, suggesting TGF-β is a key regulator of epithelial to mesenchymal transition (EMT) upon SARS-CoV-2 infection [15]. In addition to EMT, an increase in expression of α-smooth muscle actin, a differentiation marker of myofibroblasts is associated with fibrosis [16, 17]. The CXCR6/CXCL16 axis was found to have a critical role in the immunopathogenesis and pulmonary fibrosis of COVID-19, in which overexpression of their ligands promotes proliferation and collagen production [18, 19]. Besides, several interleukins, such as IL-17 and IL-25, induce proliferation and differentiation of fibroblasts, and enhance collagen synthesis as well as EMT, to promote pulmonary fibrosis [20]. Fibrosis is also characterized by an unregulated and excessive deposition of extracellular matrix (ECM) components [21]. ECM is a viscoelastic gel containing proteoglycans, hyaluronan, and various glycoproteins encompassed in a matrix of collagens, elastic fibers and fibronectin. [22]. An accumulation of ECM of viral infection induced fibrosis is typically observed [23]. Therefore, inhibition of the mentioned mediators may present a new therapeutic strategy targeting COVID-19 induced pulmonary fibrosis.

Common antiviral approaches deployed since the outbreak of COVID-19 include vaccines, cytokine-suppression, antibody-based treatments, and peptide based treatments [24, 25]. The SARS-CoV-2 spike- RBD was used as a target for antibody cocktails and an antigen for vaccine development, but common vaccines lack the ability to protect nasal tissue from SARS-CoV-2. The antiviral agents nafamostat and remdesivir [24, 26], as well as IL-6 inhibitors such as clazakizumab, siltuximab, toci-lizumab and sarilumab [5], have been commonly used. Unfortunately, untoward side effects were encountered clinically. Hepatotoxicity, gastrointestinal symptoms, respiratory toxicity, cardiovascular toxicity, nephrotoxicity and skin infections have been reported [5, 27, 28]. At present, there are no good drugs free of side effects for the therapy of post inflammatory COVID-19 lung fibrosis. The antifibrotic drugs nintedanib and pirfenidone suffer from the drawback of liver toxicity and in addition nintedanib brings about an elevated risk of bleeding since the majority of COVID-19 patients are treated with anticoagulant medications [29]. Such side effects have considerably limited the spectrum of usage of these antiviral and antifibrotic agents.

On the other hand, a positive therapeutic role of traditional Chinese medicine (TCM) has been emphasized. In China, TCM was used for treatment in about 91.5% of the confirmed COVID-19 cases [30]. A clinical efficacy rate of TCM exceeding 90% has been observed. TCM can effectively relieve symptoms and increase the cure rate. The “Diagnosis and treatment of novel coronavirus pneumonia (Trial version 8)” [31] hints that TCM possesses a potential for use in clinical medications and disease prevention and control strategies against COVID-19. At the onset of the pandemic, Ren et al*.* [32] noted that early intervention with TCM was effective in 102 COVID cases with moderate symptoms. Another study also discovered that with the use of TCM, the mean durations of fever, clinical remission, and hospital stay were all significantly reduced [33]. Up till now, an increasing amount of direct evidence based on clinical trials of TCM in the treatment of COVID-19 has been published [34]. TCM has been found to enhance the overall cure rate and attenuate the clinical manifestations of COVID-19 infected individuals. TCM may be potentially beneficial as a preventive or therapeutic anti-COVID-19 measure [33, 35, 36]. The identification of specific TCM inhibitors targeting the spike protein is also a vital approach for the prevention of COVID-19.

Allergic Rhinitis Nose Drops (ARND) is a commercially available proprietary Chinese medicine nasal spray in Hong Kong SAR, which is under the transitional arrangement of registration of proprietary Chinese medicines in Department of Health, Hong Hong SAR Government. The tests of contents of heavy metals and toxic elements conducted by Castco Testing Centre Limited, an accredited testing laboratory recognized by Department of Health, including arsenic, cadmium, lead and mercury; and residual pesticides including aldrin and dieldrin, chlordane, DDT, endrine, heptachlor, hexachlorobenzene, hexachlorocyclohexane, lindane and quintozene;and microbes including aerobic plate count, *E.coli, Pseudomonas aeruginosa,Stpaphylococcus aureus*, molds and yeasts all met the standards set by Department of Health. ARND is commonly used to treat hypersensitivity reactions in patients with allergic rhinitis [37]. There are some common upper airway symptoms in allergic rhinitis and COVID-19 [38] and hypersensitivity in the immune system, both of which involve phagocytic cells (e.g.: macrophages and monocytes) and release of cytokines (e.g.: IL-1 and TNF-α) [39]. As it is commonly used to treat airway diseases and no adverse effect has been reported, its potential utility upon SARS-CoV-2 infection and post-infection deserves investigation. In addition, ARND could exert its effects on 118 gene targets commonly affected by COVID-19 as demonstrted by us using network pharmacology analysis. Therefore, the potential effects of re-purposing ARND in suppressing SARS-CoV-2 viral infection, post-infection inflammation and fibrosis were hypothesized and examined in this study. With the use of bioinformatics investigation (network pharmacology analysis) and biotechnology validation (pseudovirus infection assay, inflammatory and fibrotic in vitro models), it may be feasible to explain the in vitro inhibitory effects of ARND on SARS-CoV-2 viral infection, cytokine storm and fibrosis.

## Results and discussion

### ARND was a potential agent against coronavirus diseases

To study if ARND was effective in targeting on genes affected by COVID-19, a network pharmacology analysis between ARND and SARS-CoV-2 was conducted. Being an extensively employed model, multi-layer networks which entail the depiction/visualization of multiple levels of interactions among medicinal plants, phytoconstituents, targets, pathways (bioprocesses), and ailments (functions, or effects) were adopted in this study [40].

#### Screening for active ingredients and related gene targets

**Fig. 1:**
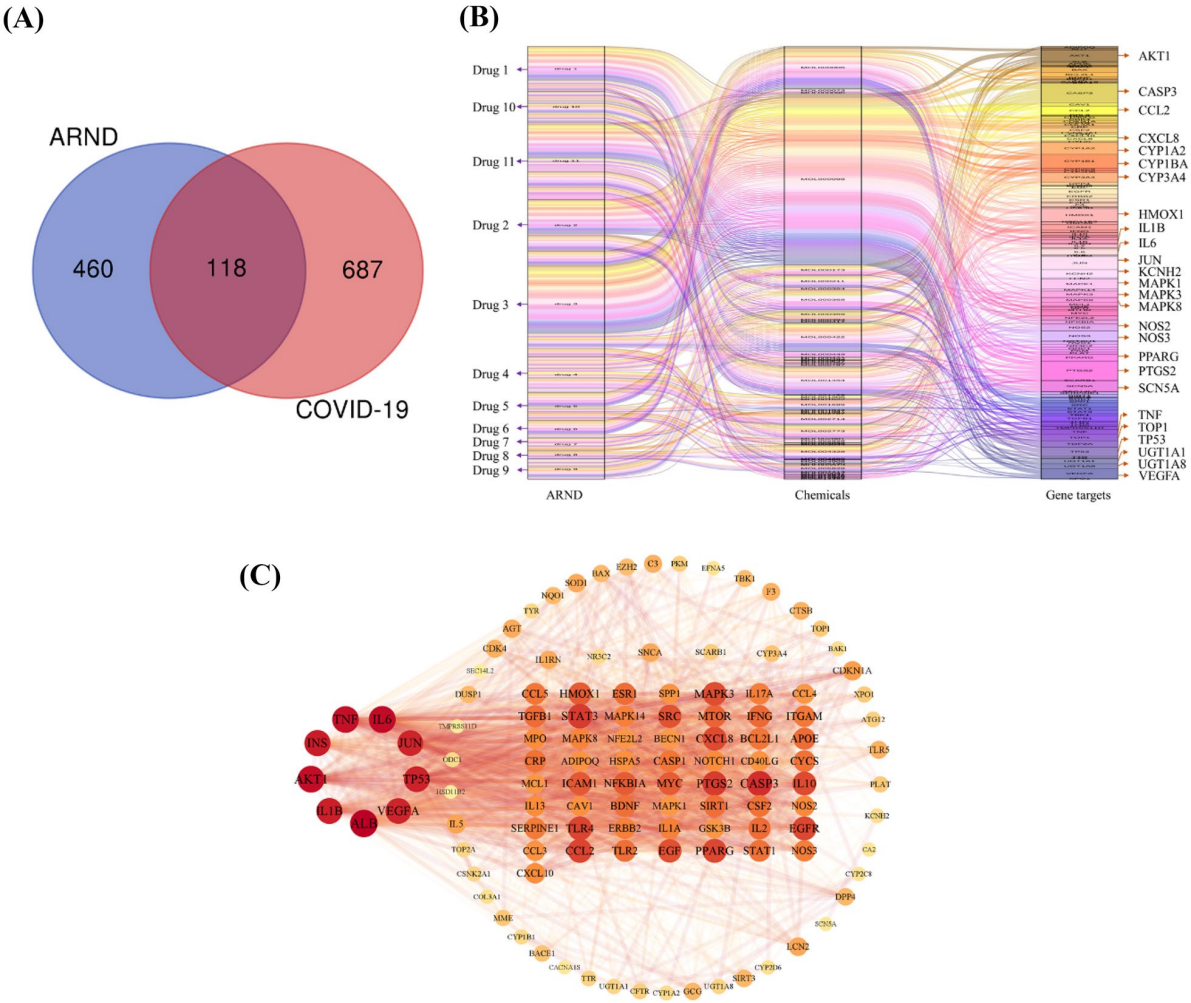
Network analysis of ARND’s targets acting on genes of the highly pathogenic and deadly human coronaviruses, namely SARS-CoV-2. **A** Venn diagram indicating intersection between ARND targeted genes (blue circle) and COVID-19 related genes (red circle). Under the condition of oral bioavailability (OB) > 30% and drug-likeness (DL) > 0.18, there were 118 gene targets commonly regulated by ARND and SARS-CoV-2. **B** Sankey diagram showing the drug–chemical-target relationships for ARND in which the height of the grid is proportional to the related score. The results showed that 118 gene targets related to COVID-19 were potentially regulated by 52 chemicals of 11 drugs in ARND.The herbs in ARND are numbered as follows. 1 = Centipedae Herba, 2 = Coptidis Rhizoma, 3 = Lonicerae Japonicae Flos, 4 = Scutellariae Radix, 5 = Menthae Haplocalycis Herba, 6 = Platycodonis Radix, 7 = Saposhnikoviae Radix, 8 = Citri Reticuulatae Pericarpium, 9 = Paeoniae Radix Alba, 10 = Glycyrrhizae Radix et Rhizoma, and 11 = Jujubae Fructus. **C** PPI network based on 118 intersected targets. The protein nodes are ordered by degree according to color from deep red (high degree) to light yellow (low degree). According to the degree, the gene targets with a degree greater than 43.864 are shown in a column chart (Additional file_METHOD_FIG_A1_TABLES_A1_12: Table A5)

For ARND, 229 compounds satisfied the criteria of an OB ≥ 30% and a DL ≥ 0.18, and 554 of their related gene targets were identified. For SARS-CoV-2, there were 805 gene targets identified (Additional file_METHOD_FIG_A1_TABLES_A1_12: Table A1). As shown in the Venn diagram in Fig. 1A and  Additional file_METHOD_FIG_A1_TABLES_A1_12: Table A1, ARND could exert its effects on 118 gene targets which were commonly affected by COVID-19. A detailed list of gene targets is shown in Additional file_METHOD_FIG_A1_TABLES_A1_12: Table A2. Since the gene targets were found related to inflammation and fibrosis, such as IL1B, NOS2, TGFB1 and IL17A, we hypothesized ARND had potential against COVID-19.

#### Drug–chemical-target and PPI network analysis

To globally depict the mechanism of ARND’s treatment to COVID-19, a drug–chemical-target relationship network, which shows 118 gene targets related to COVID-19 were potentially regulated by 52 chemicals of 11 drugs (herbs) in ARND, was constructed (Fig. 1B). The herbs are listed in the legend of Fig. 1. The most often utilized herbs for treating COVID-19 included the following as reported by different authors: Lonicerae Japonicae Flos (drug 3) [41–43], Scutellariae Radix (drug 4) [42–46], Menthae Haplocalycis Herba (drug 5) [43], Platycodonis Radix (drug 6) [42, 43], Saposhnikoviae Radix (drug 7) [42], Citri Reticuulatae Pericarpium (drug 8) [42, 44–46], Glycyrrhizae Radix et Rhizoma (drug 10) [41–43, 45, 47] which was strongly paired with Citri Reticuulatae Pericarpium [48]. Among the drugs, drugs 3, 7 and 10 were used most often for prevention of COVID-19 in China by tonifying *qi* to protect from pathogens, dispel wind, discharge heat, and eliminate dampness [42]. While drugs 4 and 8 were dampness removing herbs used to combat COVID-19 [44]. The herbs in ARND had been used in various TCM formulae effective against COVID-19 in clinical practice, such as drug 10 in Huashi Baidu Decoction; drugs 4, 8 and 10 in Qingfei Paidu decoction; drugs 6, 8 and 10 in Huoxiang Zhengqi capsule; drugs 3, 4, 5 and 10 in Jinhua Qinggan granule; drugs 3, 5 and 10 in Lianhuaqingwen capsules; drugs 3, 4 and 10 in Tanreqing injection; drug 10 in Shufeng Jiedu capsule and drug 3 in Reduning injection [49]. Herbs in ARND were also utilized in different stages of COVID-19 patients, including drug 3 frequently employed in the observational stage and drug 6 used in the mild stage; drug 4 most applied in moderate, severe and critical stages and drug 10 most utilized in all the stages [42]. The variety of clinical usage of herbs in ARND showed the potential of ARND to be re-purposed for combating COVID-19.

A detailed list of chemicals which had gene targets overlapping with those of COVID-19 and target-compound-drug list are shown in Additional file_METHOD_FIG_A1_TABLES_A1_12: Table A3, A4 respectively. The results provided information of genes and pathways that are potentially regulated by the chemical compounds in different herbs. The anti-COVID-19 chemicals in ARND and their mechanisms of anti-COVID-19 action are discussed wherever possible in the following. ACE2 and chymotrypsin like protease [3CLpro or main protease (Mpro)] are pivotal for viral entry [50] and viral replication [51] in SARS-CoV-2 infection. Herbs and chemical compounds in ARND were found to be potential inhibitors of ACE2 and 3CLpro.

Lonicerae Japonicae Flos (drug 3) was an ACE2 inhibitor [52]. Citri Reticuulatae Pericarpium (drug 8) [53] as well as quercetin and glabridin from Glycyrrhizae Radix et Rhizoma (drug 10) [54] were able to downregulate ACE2. Oroxylin A from Scutellariae Radix (drug 4) demonstrated binding to the ACE2 receptor on human embryonic kidney (HEK293T) cells and prevented SARS-CoV-2 from entering the cells [55]. Paeoniae Radix Alba (drug 9) is a 3CLpro inhibitor and it contains kaempferol and beta-sitosterol both of which inhibit SARS-CoV2 [56]. Berberine from Coptidis Rhizoma (drug 2) was also reported to have potential inhibitory activity on COVID-19 Mpro [57]. It further downregulates pro-inflammatory cytokines, prevents SARS-CoV-2 infection and replication [58]; and exerts a protective action against tissue damage [59]. Beta-sitosterol is found in many herbs, including Platycodonis Radix (drug 6), drugs 3 and 10. It exerts potential inhibitory activity on SARS-CoV2 Mpro [60]. Indeed, multiple chemical compounds in ARND targeted on either ACE2 or 3CLpro, such as kaempferol, luteolin and quercetin from Lonicerae Japonicae Flos (drug 3) [61], baicalein, wogonin and oroxylin A from Scutellariae Radix (drug 4) [62, 63] and naringenin from Citri Reticuulatae Pericarpium (drug 8) [64]. They manifested anti-inflammatory, immunomodulatory, and free radical scavenging activities through their actions on a multitude of molecules encompassing (Caspase 3) CASP3, IL-6, and mitogen-activated protein kinase (MAPK) 1, 8, and 14, in the signaling pathways of IL-17, NF-κB, TNF and NOS [63, 65, 66] to achieve their anti-COVID-19 mechanisms.

Among the chemical compounds, luteolin showed high affinity binding to SARS-CoV-2 Mpro [67]. Kaempferol showed high affinity binding at the substrate binding pocket of 3CLpro and interacted with the active site residues comprising His41 and Cys145 through hydrophobic interactions and hydrogen bonding [66]. Quercetin inhibited 3CLpro, papain-like protease (PLpro), and SARS-CoV-2 replication, and manifested anti-inflammatory and thrombin-inhibitory activities [68]. Baicalein and the ethanolic extract of drug 4 suppressed SARS-CoV-2 replication [69] in Vero cells with an EC_50_ value of 2.9 µM and 0.74 µg/ml, respectively and SARS-CoV-2 3CLpro with an IC_50_ value of 0.39 µM and 8.52 µg/ml, respectively [62]. Baicalein also demonstrated high affinity binding to transmembrane serine protease 2 (TMPRSS2), another key player in cellular entry by the SARS-CoV viruses with Asp-345, His-296 and Ser-441 in the active binding site [70].

In addition to Additional file_METHOD_FIG_A1_TABLES_A1_12: Table A3, there are also other chemical compounds in ARND with reported effects on ACE2 or 3CLpro against COVID-19. For example, hesperidin from Citri Reticuulatae Pericarpium (drug 8) forestalls SARS-CoV-2 entry to the host through ACE2 receptors. It also has anti-inflammatory activity for alleviating cytokine storm. A mixture of hesperidin and diosmin when given in conjunction with heparin protected against venous thromboembolism and thus slows disease deterioration [71, 72]. Glycyrrhizin from Glycyrrhizae Radix et Rhizoma (drug 10) brought about cholesterol-dependent lipid raft disorganization paramount to coronaviruses to gain entry into cells. At levels found inside the cells and in the circulation, glycyrrhizin sequestered high mobility group box 1 protein and interfered with its action as alarmin [73]. Drug 10 extract and glycyrrhizin impeded uptake of COVID-19 into host cells, thwarted the interaction between ACE2 and receptor-binding domain of SARS-COV2, and exerted protective actions against inflammation-induced acute pulmonary damage and cardiovascular derangements [74]. A patient who experienced severe COVID-19 and treated with steroid-like diammonium glycyrrhizinate together with ascorbic acid was able to recover from the disease [75]. Network pharmacology followed by molecular docking were employed to ascertain the mechanism through which chlorogenic acid from Jujubae Fructus (drug 11) [76] affected COVID-19, resulting in 70 potential targets associated with COVID-19 treatment, with ACE, estrogen receptor 1 (ESR1), heme oxygenase 1 (HMOX1), IL-6, and NFE2 Like BZIP transcription factor 2 (NFE2L2) and peroxisome proliferator activated receptor gamma (PPARG), as the key potential targets. The potential anti-SARS-CoV-2 activity of chlorogenic acid was exerted through integrating three common receptors in clinical practice in comparison with clinical trial drugs registered for COVID-19 treatment, as demonstrated by molecular docking [77]. Chlorogenic acid which exhibits binding affinity to cell-surface heat shock protein A5 substrate-binding domain β, the SARS-CoV-2 spike protein recognition site, would impair SARS-CoV-2 attachment to host cells [78]. Ursolic acid and ursonic acids which are triterpenes from drug 11 [76, 79] were shown to be potential inhibitors of the Mpro of COVID-19 [79–81]. Glycyrrizin from drug 10 [57] and hesperetin from Citri Reticuulatae Pericarpium (drug 8) [57] were also found to possess potential inhibitory activity on COVID-19 Mpro.

Moreover, the action mechanisms of reported chemical compounds in ARND combating COVID-19 were diverse. Pathway analysis indicated that baicalin (drug 4) has targets in human cells associated with signals of pro-inflammatory cytokines [82]. The results of prediction by using the Swiss Target Prediction server disclosed that norwogonin (drug 4) and baicalein displayed binding with a high affinity for enzymes associated with pulmonary damage, such as arachidonate 15-lipoxygenase (ALOX15), cycline dependent kinase 1 (CDK1), lysine-specific demethylase 4D (KDM4D), and xanthine dehydrogenase (XDH) [63]. The triterpenoid saponin platycodin D from Platycodonis Radix (drug 6) obstructed the two major routes of SARS-CoV-2 infection through lysosome- and TMPRSS2-driven entrance. Platycodin D forestalled SARS-CoV-2 entry to the host by redistributing membrane cholesterol to repress membrane fusion, which could be reinstated by administration of an agent encapsulating platycodin D [83]. Results from the network pharmacology study disclosed the presence of a multitude of compounds in ARND with possible repressive activity on SARS-CoV-2. The results also provided a basis for repurposing ARND and potential compounds in ARND to be deployed as COVID-19 inhibitors.

To select the key targets between ARND and SARS-CoV-2 for further verification, the 118 intersection targets were analyzed with STRING and imported into CytoScape for construction of a PPI network (Fig. 1C). The protein nodes were arranged in order by degree according to the colour from deep red (high degree) to light yellow (low degree). The degrees of 66 nodes were greater than the average number of neighbors (43.864) and were coloured from as deep red to orange (Additional file_METHOD_FIG_A1_TABLES_A1_12: Table A5). Among the identified gene targets, IL6, TNF, IL1B, CCL2, NFKBIA, NOS2, TGFB1 and IL17A, which are respectively related to inflammation and fibrosis and detected at heightened levels in COVID-19 patients, were selected for further validation of the network pharmacology results.

#### Enrichment analysis

**Fig. 2:**
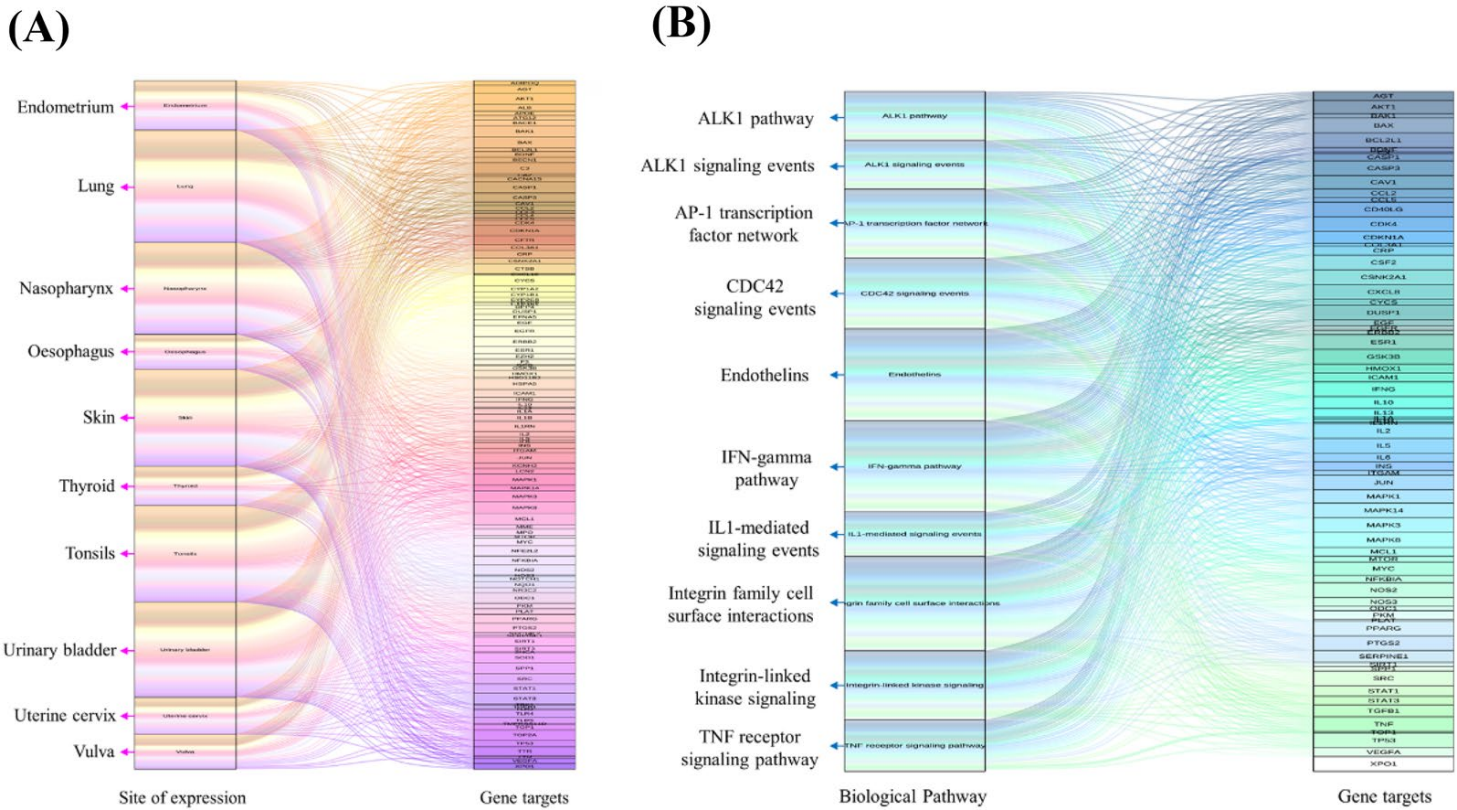
Site of expression and biological pathway enrichment analysis of ARND. According to the results, **(A)** lung takes the highest percentage of genes between ARND and COVID-19 for site of expression and **(B)** the common biological pathways between ARND and COVID-19 was integrin family cell surface interactions. The height of the grid is proportional to the number of genes

For the site of expression enrichment analysis, the *p*-value of gene targets and top ten results analyzed by the Funrich software are summarized in Fig. 2A and Additional file_METHOD_FIG_A1_TABLES_A1_12: Table A6. According to the results, the lungs are involved with the highest percentage of genes between ARND and COVID-19. The lungs are the organs most adversely impacted by COVID-19 because the SARS-CoV-2 virus attaches to host cells via ACE2, which occurs in abundance on the surface of type II alveolar cells in the lungs. The common biological pathways which ARND and SARS-CoV-2 affect were integrin family cell surface interactions (Fig. 2B and Additional file_METHOD_FIG_A1_TABLES_A1_12: Table A7). Viruses bind to cell-surface integrins facilitating viral entry, whereas integrins mediate a variety of signaling pathways which are dysregulated by SARS-CoV-2 virus binding, leading to tissue damage [84]. Results pertaining to integrins suggest that ARND acts by inhibiting viral interactions with the integrins to exert its antiviral effect.

**Fig. 3:**
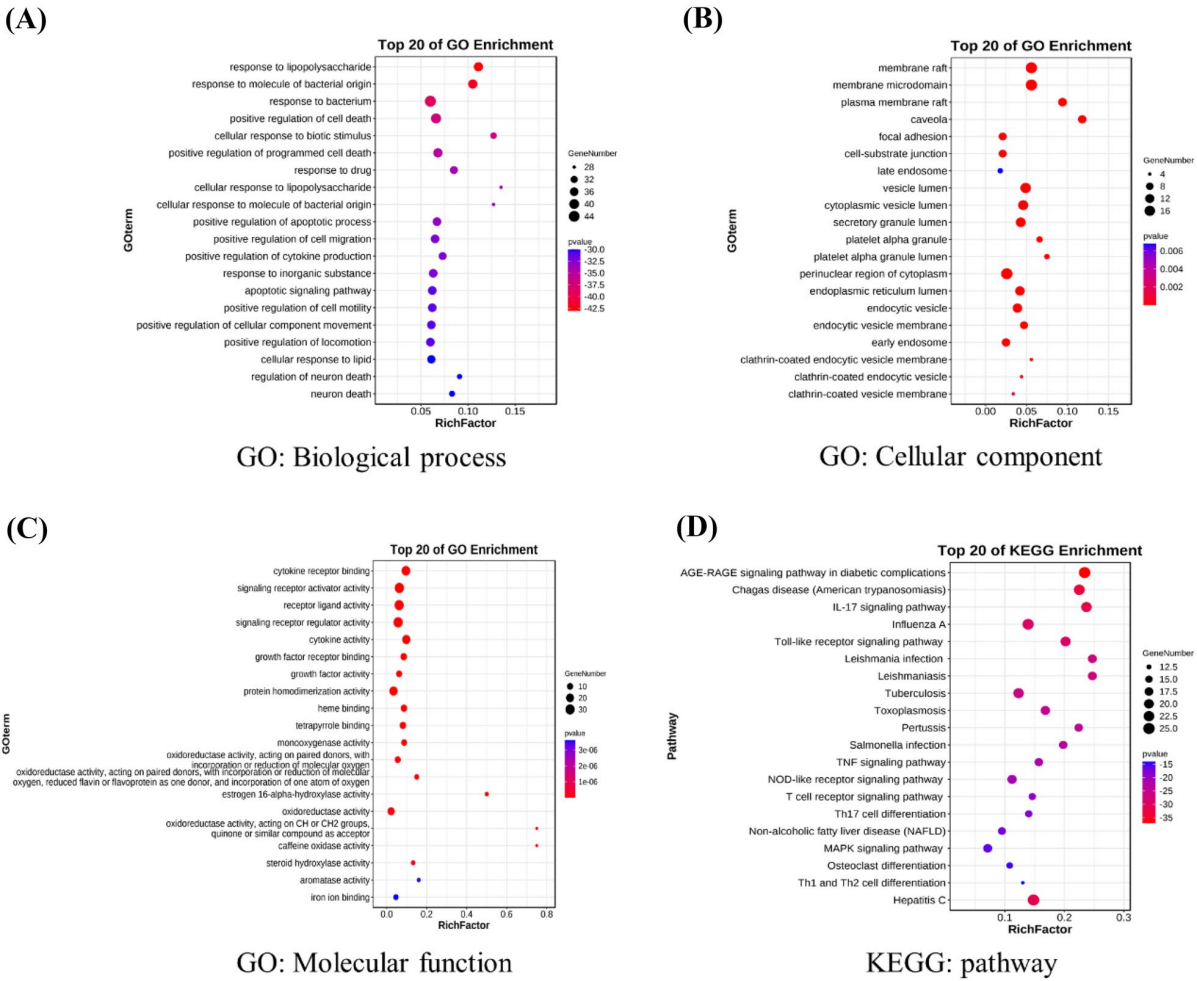
GO and KEGG enrichment analysis of ARND. Bubble charts of **(A)** biological process, **(B)** cellular component and **(C)** molecular function from GO enrichment analysis as well as **(D)** signaling pathways from KEGG enrichment analysis. ARND was found to be closely related to the response to lipopolysaccharide, membrane rafts and cytokine receptor binding as well as AGE-RAGE signaling pathway in diabetic complications. The x-axis and y-axis showed gene ratios and full names of the category of results, respectively. The color and size of each bubble represent the adjusted *p*-value and gene count, respectively

Through GO enrichment analysis based on common ARND-COVID-19 gene targets, a total of 2345 terms related to the effects of ARND on COVID-19 were obtained, and these terms could be divided into three categories, including 2150 terms of biological process, 66 terms of cellular component and 129 terms of molecular function. The top 20 terms in the three categories above are shown as bubble charts in Fig. 3A–C and Additional file_METHOD_FIG_A1_TABLES_A1_12: Tables A8–10. According to the results, ARND primarily targeted the response to lipopolysaccharide, membrane rafts and cytokine receptor binding. This suggested that ARND could activate or halt gene targets and/or cytokines related to the lipopolysaccharide-mediated signaling pathways [85] and viral entry through membrane rafts [86], hence altering the actions of COVID-19.

Through the results of KEGG pathway enrichment analysis, we acquired 166 signaling pathways involved in the possible mechanism by which ARND affects COVID-19 and the top 20 pathways are shown in Fig. 3D and Additional file_METHOD_FIG_A1_TABLES_A1_12: Table A11. The results suggested that the intersection targets are concentrated mainly in AGE-RAGE signaling pathway in diabetic complications. It has been known that increased levels of advanced glycation end products (AGEs) are associated with diabetes and receptor for advanced glycation end products (RAGE) is mainly expressed by type II alveolar cells, which has a significant role in SARS-CoV-2 induced cytokine storm and lung injury [87]. Besides, the accumulation of AGEs is also found during aging, this may add value to why aging is one of the risk factors for COVID-19 [87]. These results altogether supported that ARND may have potential against COVID-19 by acting on the AGE-RAGE signaling pathway and it may be pertinent especially for the elderly.

### ARND significantly suppressed pseudovirus infection by interrupting viral entry via ACE2

**Fig. 4:**
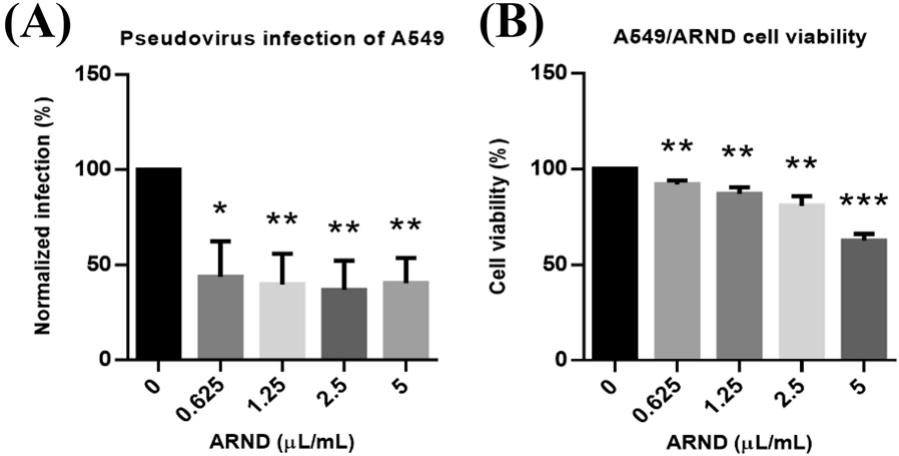
ARND prevented SARS-CoV-2 pseudovirus infection of A549 lung cells. **A** A549 cells were pre-treated for 2 h with different concentrations of ARND followed by pseudovirus infection for 3 h (n = 4). Green fluorescent protein signal indicative of infection was examined and analyzed. ARND at all concentrations tested inhibited SARS-CoV-2 pseudovirus infection in A549 cells and **(B)** A549 cells were treated for 24 h with different concentrations of ARND (n = 4). The absorbance was measured with a microplate reader. ARND at all concentrations did not elicit > 50% reduction in viability of A549 cells. *p < 0.05, **p < 0.01 and ***p < 0.001 vs 0 µL/mL (0.5% DMSO)

To screen for anti-viral effects of drugs or agents, a pseudovirus infection model was typically employed [88]. Pseudoviruses are good surrogates for the extremely hazardous and pathogenic SARS-CoV-2 virus owing to genetic stability, scalability and safety for performing assays to screen for drug candidates [89]. In this study, a SARS-CoV-2 pseudovirus infection model with upper respiratory tract epithelial A549 cells or with transient ACE2-overexpressed 293 T cells was employed [90, 91]. It was revealed that, after pre-treatment with ARND at concentrations ranging from 0.625 to 5 µL/mL, a significantly (*p* < 0.05) abated percentage (> 50%) of pseudovirus infection was detected in ARND-treated groups versus the control group without ARND treatment (Fig. 4A). Besides, in Fig. 4B, the reduction in viability of ARND-treated A549 cells did not exceed 50%, indicating that ARND did not affect lung cell viability. The decreased infection percentage was not an outcome of a decreased number of cells but rather the consequence of the action of ARND. Figure 4 demonstrates that ARND exerted a suppressive action on pseudovirus infection of the human lung A549 cells, which corroborated the results in network pharmacology analysis that ARND had potential antiviral activity.

**Fig. 5:**
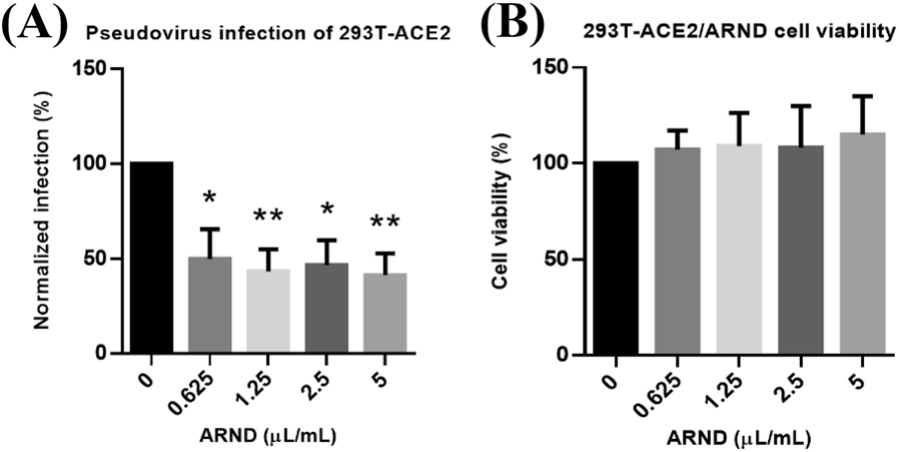
ARND prevented SARS-CoV-2 pseudovirus infection of 293 T-ACE2 cells. **(A)** 293 T-ACE2 cells were first exposed for 2 h to ARND at different concentrations followed by pseudovirus infection for 3 h (n = 3). Green fluorescent protein signal indicative of infection was examined and analyzed. ARND at all concentrations tested inhibited SARS-CoV-2 pseudovirus infection of 293 T-ACE2 cells. **(B)** 293 T-ACE2 cells were treated for 24 h with different concentrations of ARND (n = 3). The absorbance was determined with a microplate reader. ARND at all concentrations did not elicit > 50% reduction in cell viability of 293 T-ACE2 cells. *p < 0.05, **p < 0.01 vs 0 µL/mL ARND (0.5% DMSO)

A previous study revealed that ACE2 binding was one of the mechanisms of cell entry during SARS-CoV-2 infection [50]. Hence, interrupting the virus receptor binding of ACE2 in the human body may prevent and control the infection of SARS-CoV-2 [92]. To confirm if the inhibitory effect of ARND on pseudovirus infection was achieved by interrupting viral entry via ACE2, transient transfection of 293 T cells for overexpressing ACE2 followed by pseudovirus infection was performed. In Fig. 5A, the results suggested ARND exerted a restraining effect of more than 50% on the percentage of infection (*p* < 0.05) when ACE2 was overexpressed in 293 T cells. In Fig. 5B, the viability of ACE2 overexpressing 293 T cells treated with different concentrations of ARND all exceeded 50%, indicating that the viability of the cells was not affected by ARND. The results in Fig. 5 revealed that ARND prevented pseudovirus infection by interrupting viral entry through ACE2, which validated the hypothesis generated in network pharmacology analysis that ARND may act on genes related to viral entry. The results also provided evidence for further validation with live virus.

### ARND interrupted viral entry by blocking Spike RBD-ACE2 binding

**Fig. 6:**
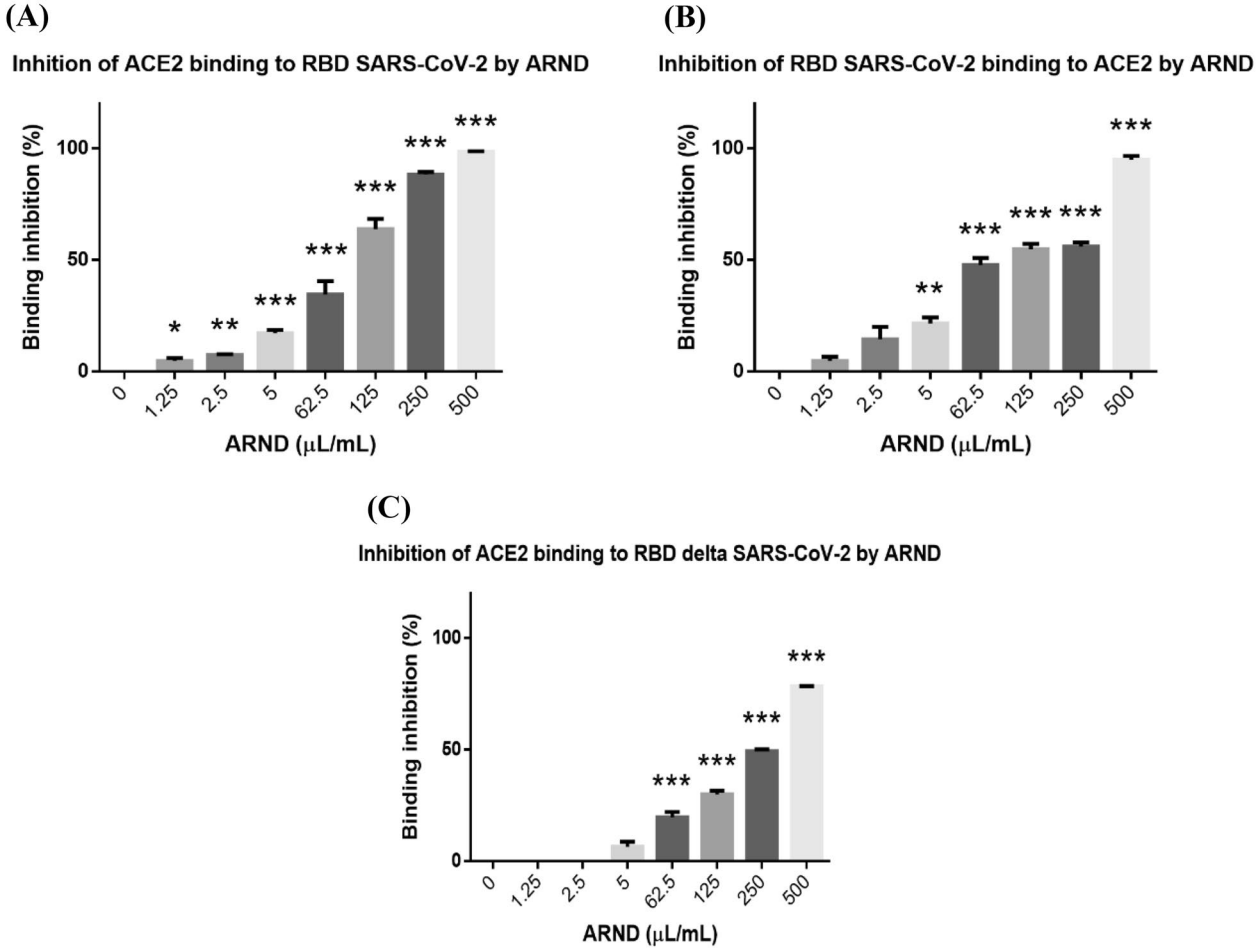
ARND could bind to either ACE2 or Spike RBD. **A** ARND at different concentrations was able to bind to ACE2 receptor, and significantly competed with the Spike RBD in formation of the Spike RBD-ACE2 complex (n = 3). **B** ARND at different concentrations was also able to dose-dependently bind to Spike RBD. The higher the concentration of ARND, the higher was the percentage inhibition of binding (n = 3). *p < 0.05, **p < 0.01 and ***p < 0.001 vs 0 µL/mL

Besides ACE2 receptor, RBD of the spike protein, a glycoprotein on the viral surface, is the most important domain of coronaviruses in the process of virus-receptor interaction for cell entry [93]. To specify the target of ARND in interfering with viral entry, the assay of inhibition of formation of the Spike RBD-ACE2 complex was performed, in which the effects of ARND on competing for binding to ACE2 or Spike RBD were examined. In Fig. 6A, the results revealed that ARND at both low concentrations (1.25–5 µL/mL) and high concentrations (62.5 to 500 µL/mL) was able to bind to ACE2 receptor in a dose-dependent manner, and very significantly (*p* < 0.001) competed with the Spike RBD in the formation of the Spike RBD-ACE2 complex. The results in Fig. 6B disclosed that ARND at different concentrations was also able to dose-dependently compete for binding to Spike RBD (*p* < 0.001). Both results suggested that viral entry could be hindered by ARND, which bound to either ACE2 or Spike RBD, in blocking the binding of ACE2 and Spike RBD and thus impeding formation of Spike RBD-ACE2 complex (Fig. 6A, B). Previous surface plasmon resonance assay with glycyrrhizic acid, and molecular docking study of baicalin and glycyrrhizin suggested that they exerted inhibitory activity against the Spike RBD of SARS-CoV-2 and/or ACE2 of host receptor by docking [93, 94]. A recent patent disclosed that Centipeda minima (drug 1 in ARND) acts on ACE2 [110]. As ARND may contain these chemical compounds, this may explain why ARND has a potential in preventing SARS-CoV-2 infection.

The SARS-CoV-2 virus has mutated in the spike region over time [95, 96], resulting in a stronger association with human ACE2 (hACE2) and a higher infectivity than the original strain [97]. To study if ARND was a potential inhibitor of SARS-CoV-2 variants, its inhibitory effect on delta Spike RBD with a commercial available kit was studied. In Fig. 6C, ARND was found to significantly (*p* < 0.001) compete with the delta Spike RBD in the formation of the Spike RBD-ACE2 complex at both low concentrations (1.25–5 µL/mL) and high concentrations (62.5 to 500 µL/mL) in a dose-dependent manner. The above results (Fig. 4, 5, 6) constitute the first report on the effects of a commercially available TCM composition, ARND, in thwarting pseudoviral infection via interrupting viral entry through ACE2. Different drugs and agents have been re-purposed for combating COVID-19, and they have different mechanisms of action against SARS-CoV-2 [98], For instance, remdesivir is known to inhibit viral replication upon infection [99]. In the present study, ARND has been re-purposed to have potential for acting against COVID-19 by interfering with viral entry of SARS-CoV-2.

### ARND attenuated inflammatory response of RAW 264.7 cells

**Fig. 7:**
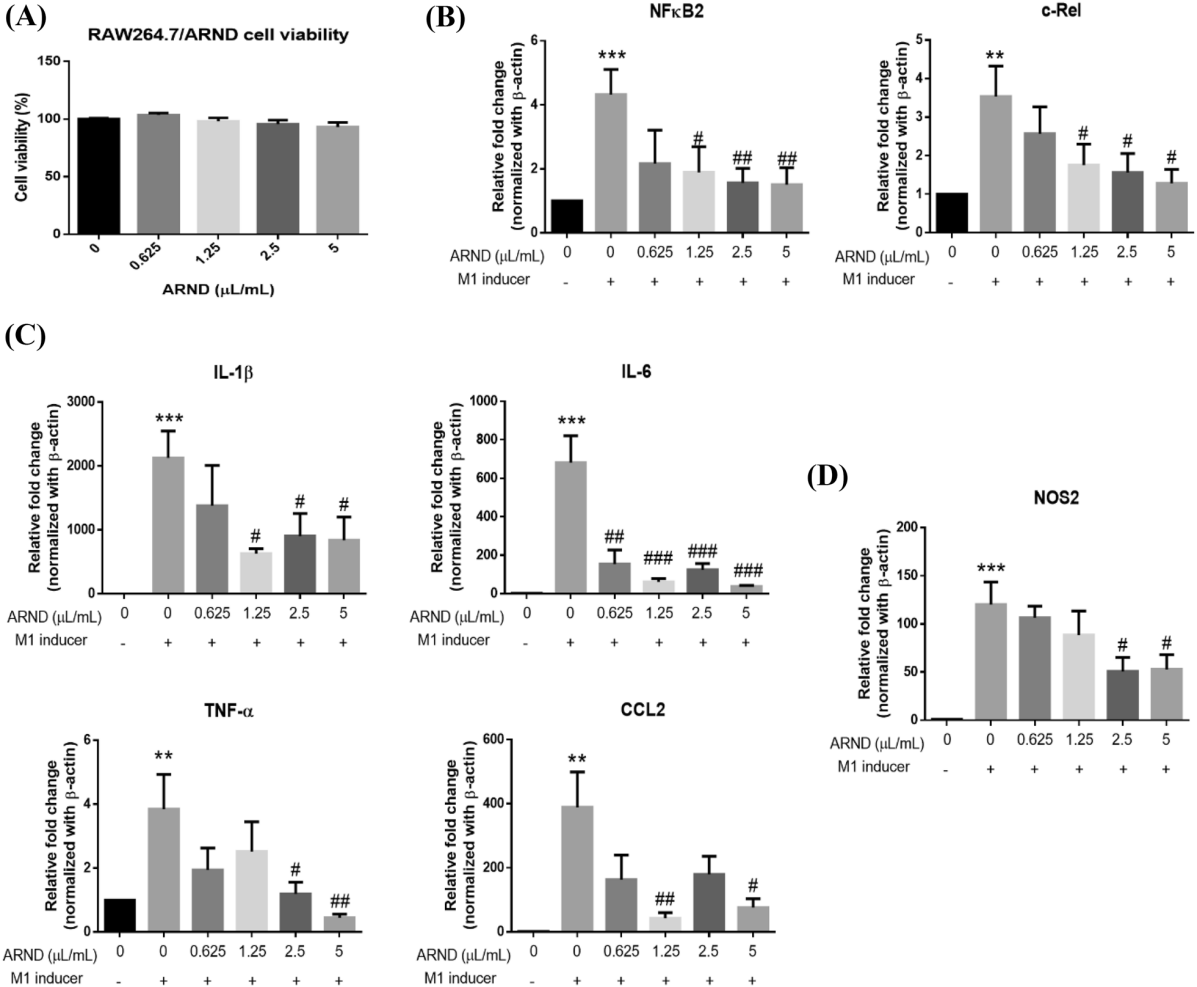
ARND exhibited anti-inflammatory effects. **A** Viability of RAW264.7 macrophages was unaltered after treatment with 0.625–5 µL/mL ARND for 48 h, suggesting that ARND was devoid of toxicity (n = 5). **B–D** Treatment with inflammatory M1 inducers (20 ng/mL IFN-γ and 100 ng/mL LPS) was used to establish the inflammatory cell model. The group receiving neither ARND nor inflammatory M1 inducers served as the control group whereas the group treated with only inflammatory M1 inducers served as the model group. The mRNA expression levels of the inflammatory biomarkers, including transcription factors (NFκB2 and c-Rel), cytokines (IL-1β, IL-6, TNF-α and CCL-2) as well as nitric oxide synthase 2 (NOS2) were significantly upregulated in the model group, while cells pre-treated with ARND showed significant downregulation of the mRNA expressions of the inflammatory biomarkers (n = 4). * *p* < 0.05, **p < 0.01 and ***p < 0.001 vs 0 µL/mL

To assess the anti-inflammatory effect of ARND, RAW264.7 macrophages were employed. M1 macrophages are defined as macrophages secreting pro-inflammatory cytokines and involved in different inflammatory processes [7]. Therefore, typical inflammatory M1 inducers, namely LPS and IFN-γ were used. The results of the cell viability assay showed that ARND did not inhibit cell proliferation from 0.625 to 5 µL/mL, suggesting that ARND was devoid of toxicity (Fig. 7A). After treatments with ARND and inflammatory M1 inducers (20 ng/mL IFN-γ and 100 ng/mL LPS), mRNA expression levels of transcription factors (NFκB2 and c-Rel), cytokines (IL-1β, IL-6, TNF-α and CCL-2) as well as nitric oxide synthase 2 (NOS2) in RAW264.7 cells were measured to examine the effects of ARND on the inflammatory response. The group which did not receive treatment with either ARND or inflammatory M1 inducers served as the control group. The group receiving treatment with only inflammatory M1 inducers served as the model group. It was demonstrated that a significant increment in mRNA expression levels of NFκB2, c-Rel, IL-1β, IL-6, TNF-α, CCL-2 and NOS2 occurred in the model group (*p* < 0.01). The mRNA expression levels of the inflammatory biomarkers, including those selected from network pharmacology analysis, were significantly undermined after treatment with ARND (*p* < 0.05), signifying that in line with results from network pharmacology analysis, ARND manifested an anti-inflammatory effect (Fig. 7B–D). The majority of herbs in ARND, such as Lonicerae Japonicae Flos, Glycyrrhizae Radix et Rhizoma and Jujubae Fructus, are known to elicit different extents of anti-inflammatory effects, [100]. There are also common chemical components in herbs of ARND, such as quercetin and kaempferol targeting multiple genes related to inflammation (Additional file_METHOD_FIG_A1_TABLES_A1_12: Table A4). This has led to the results that ARND possessed anti-inflammatory activity, which may be useful for suppressing the cytokine storm brought about by infection with SARS-CoV-2. The above results also supported further investigations on the possible ability of ARND to inhibit inflammation in vivo.

### ARND prevented fibrosis of Calu-3 cells

**Fig. 8:**
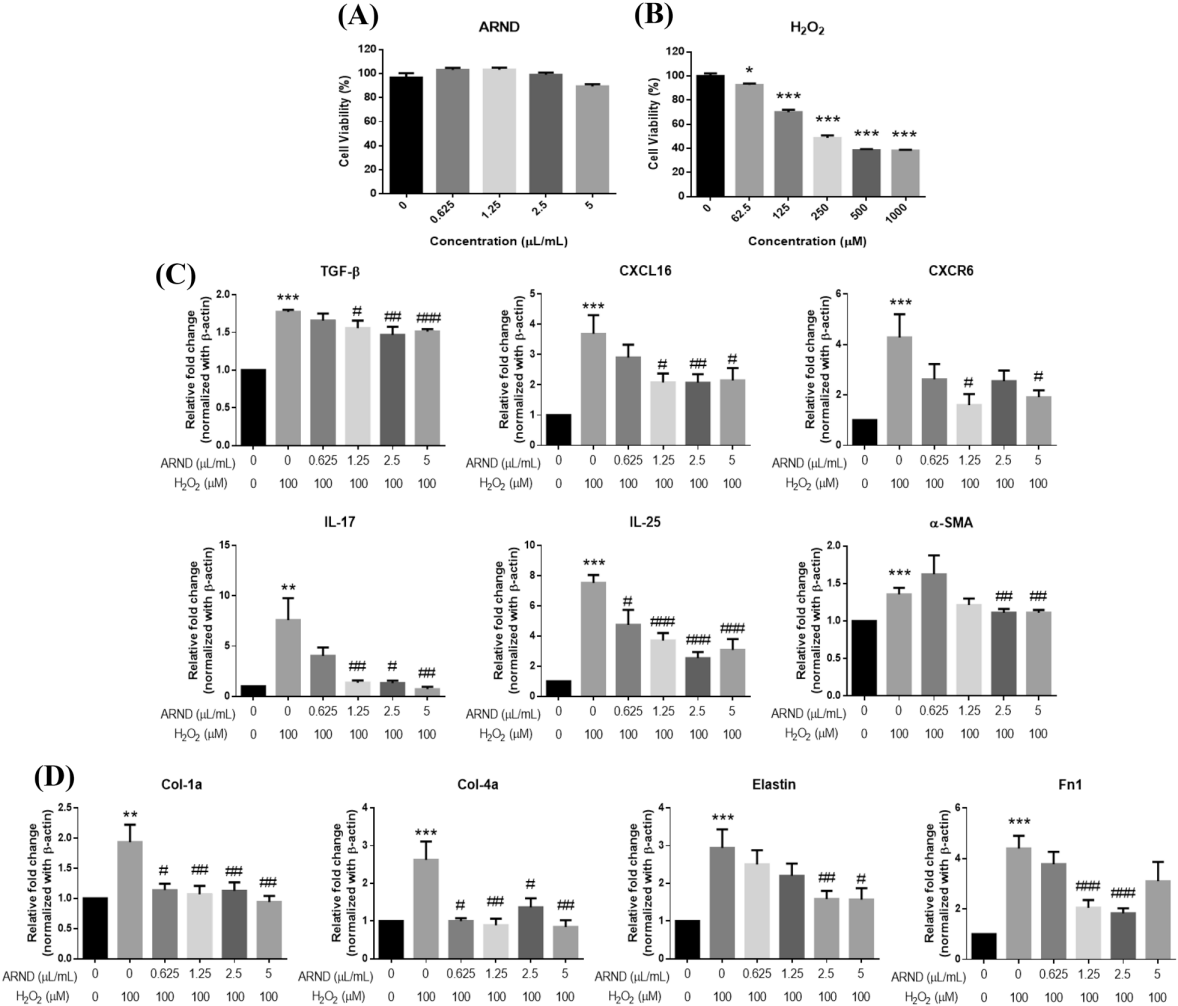
ARND manifested an anti-fibrotic effect. **A** Calu-3 cells were treated with different concentrations of ARND (0.625–5 µL/mL) for 48 h. Treatment with different concentrations of ARND did not reduce viability in Calu-3 cells, indicating ARND manifested no substantial toxicity towards Calu-3 cells. (n = 4). **B** Calu-3 cells were exposed to different concentrations of H_2_O_2_ for 24 h. A decline in cell viability was observed at H_2_O_2_ concentrations between 62.5 (*p* < 0.05) and 125 µM (*p* < 0.001). Therefore, the approximate mean of the two concentrations i.e., 100 µM H_2_O_2_ was selected for establishing the cell model of lung fibrosis (n = 3). *p < 0.05 and ***p < 0.001 vs 0 µM. **C–D** The mRNA expression levels of 10 biomarkers related to epithelial-mesenchymal transition (EMT; TGF-β, CXCL-16, CXCR4, IL-17, IL-25 and α-SMA) as well as extracellular matrix (ECM; Col-1a, Col-4a, elastin, and Fn1) of fibrosis were examined after ARND pre-treatment and co-treatment with H_2_O_2_. The group without receiving any treatment served as the control group while the group with only H_2_O_2_ (100 µM) treatment served as the model group. There was a significant upregulation of mRNA expression levels of the 10 fibrotic biomarkers in the model group (*p* < 0.05), while cells co-treated with ARND demonstrated a significant downregulation of the mRNA expressions levels of different fibrotic biomarkers, albeit to different extents (*p* < 0.05). (n = 4). **p < 0.01 and ***p < 0.001 vs the control group; ^#^*p* < 0.05, ^##^*p* < 0.01 and ^###^*p* < 0.001 vs the model group

Previous studies disclosed that the hyperinflammatory phenotype was considerably higher in the bronchi than in the nasopharynx of the COVID-19 patients [101, 102]. Oxidative stress in the lung tissues is a characteristic of infections caused by SARS-CoV-2 [103] and an important mechanism underlying fibrosis [104]. To study if ARND could prevent fibrosis, a H_2_O_2_-induced lung fibrosis cell model of the lower respiratory tract Calu-3 cells was established for investigation [105, 106]. Before establishment of the fibrotic cell model, the effects of ARND and H_2_O_2_ on cell viability of Calu-3 cells were examined. ARND at different concentrations (0.625–5 µL/mL) were used to treat Calu-3 cells. The results suggested that ARND did not reduce viability of Calu-3 cells, demonstrating that ARND was devoid of toxicity towards Calu-3 cells (Fig. 8A). Different concentrations of H_2_O_2_ were also studied to select the appropriate concentration for further experiments. In Fig. 8B, it was observed that a significant decrease in cell viability was detected at H_2_O_2_ concentration from 62.5 (*p* < 0.05) to 125 µM (*p* < 0.001). Thus, the approximate mean value of these two concentrations of H_2_O_2_ (100 µM) was selected for establishment of the model of cell fibrosis.

After co-treatment with ARND and H_2_O_2_, the mRNA expression levels of different fibrotic biomarkers related to EMT (TGF-β, CXCL-16, CXCR4, IL-17, IL-25 and α-SMA) as well as ECM (Col-1a, Col-4a, elastin, and Fn1) were examined. A significant rise in expression levels of the biomarkers in the model group with only H_2_O_2_ treatment (100 µM) was observed (*p* < 0.05) versus the control group without H_2_O_2_ treatment (0 µM). In those groups receiving pre-treatment with different concentrations of ARND, a significant decline to various extents was observed in expression levels of those biomarkers. The results in Fig. 8C suggested ARND significantly reduced the expression levels of EMT biomarkers, including TGF-β and IL-17 selected from network pharmacology analysis (*p* < 0.05), indicating its potential effect of reversing the EMT state of the cells. In Fig. 8D, ARND significantly ameliorated the upregulation in expression levels of Col-1a, Col-4a, elastin, and Fn1 (*p* < 0.05), suggesting ARND could remodel the ECM in the lung tissue to prevent fibrosis. ARND inhibited the mentioned mediators which supported further investigation of it being a therapeutic agent targeting pulmonary fibrosis. A previous study suggested that chemical compounds such as quercetin was effective for pulmonary fibrosis therapy [107] and quercetin was one of the chemical components in ARND (Additional file_METHOD_FIG_A1_TABLES_A1_12: Table A4), which has been studied in a clinical trial for its beneficial effects against COVD-19 at an early stage [108]. Results from the present study lay foundation for further animal studies and clinical trials.

ARND used in the present study complies with the international requirements for limits regarding microbial contaminants, and pesticide and heavy metal residues. In a published randomized, placebo-controlled and double-blinded study [37], with a cross-over arrangement for placebo or ARND administration, patients with clinically confirmed perennial allergic rhinitis were randomly allocated to two groups: an ARND-treated group and a placebo-treated group,with 20 and 15 patients in the 2 groups respectively. The ARND group received ARND (2 sprays per nostril, 5 times daily) in weeks 0–2, washout in weeks 3–5 and placebo in weeks 4–6. The placebo group received placebo in weeks 0–2, washout in weeks 3–5 and ARND in weeks 4–6. The patients were assessed by an internal medicine specialist and also examined by a Chinese medicine practitioner based on the Chinese medicinal practice. Blood analysis and assessment of quality of life were performed at baseline, and again at the end of the 2nd, 5th and 7th weeks. The Clinical Symptoms Scores of the patients based on their rhinitis symptoms i.e. nasal itching (itchiness), nasal obstruction (stuffiness), running nose, and sneezing, where 0 = lack of symptoms; 1 = slight symptoms; 2 = moderate symptoms; and 3 = severe symptoms, with the total score from 0 to 12 for the four types of symptoms were recorded by the internal medicine specialist. The scale of scores was based on the study protocol of Ventura et al. [109] on fluticasone. Laboratory tests of hematological status (complete blood picture such as erythrocytes and leukocytes), fasting glucose, C-reactive protein, alanine aminotransferase reflecting hepatic function, and creatinine reflecting kidney function were performed. The change in quality of life of the patients was determined with the instrument ChQOL designed by the Research and Development Division, School of Chinese Medicine, Hong Kong Baptist University [110]. Alleviation of symptoms, i.e., nasal itching (itchiness), nasal obstruction (stuffiness), running nose, and sneezing evidenced by reduction in the total Clinical Symptoms Score values was noted in almost all of the patients after ARND treatment at the end of the 2nd week in the ARND- treated group and at the end of the 7th week in the placebo- treated group. Improvements were limited to sneezing and running nose in the ARND- treated group after placebo treatment. In contrast, no symptomatic changes were detected at the end of the 2nd week in the placebo- treated group after placebo treatment. The results revealed the absence of any adverse alterations in liver and kiney functions, C-reactive protein and haematological parameters throughout the course of the investigation. In the ARND-treated group, significantly better sleep and complexion were noted following ARND treatment, but not following placebo treatment when the scores were compared with those obtained after the washout period. A significant reduction of consciousness and spirit of the eye bringing about a significant decline in the score of the domain of spirit was noted following placebo treatment in the ARND-treated group, but not following ARND treatment. In the placebo-treated group, no significant changes in any aspect was seen when the patients received placebo treatment for the first two weeks. However, significantly better appetite and digestion and pleasure were noticed due to ARND treatment subsequent to the washout period. When the results from the ARND-treated group after ARND treatment in the first 2 weeks were pooled with those from the placebo-treated group after ARND treatment in the last 2 weeks, significantly better sleep and complexion were seen when the ARND treatment was completed in comparison with the total baseline level. No changes were seen when results from the ARND-treated group were pooled with those from the placebo-treated group after the placebo treatment. The above results illustrated that ARND is safe. No reports on undesirable reactions have appeared over the years since its launching in the market. The present report demonstrates its additional potential usefulness to prevent SARS-CoV-2 coronavirus infection. The clinical efficacy of ARND against the SARS-CoV-2 coronavirus and its various active principles disclosed in previous investigations mentioned in “Drug-chemical-target and PPI network analysis” section above remain to be established. The multiplicity of active ingredients in Chinese medicinal formulas [42–49]and western drugs [111, 112] composed of ingredients with distinctly different modes of action used to combat SARS-CoV-2 coronavirus infection is a common observation.

In a nationwide study performed in Korea, allergic rhinitis brought about an elevated risk of susceptibility to COVID-19 infection and poorer prognosis of COVID-19 [113]. In contrast, in a study carried out in Turkey [114] and in another conducted in Italy [115], the severity of COVID-19 was not influenced by allergic rhinitis.Pediatric allergic rhinitis may be accompanied with a milder course of COVID-19 disease [116]. In Chinese patients, allergic rhinitis did not affect COVID-19 mortality. Anti- allergic rhinitis drugs including antihistamines, β2 adrenoceptor agonists,and corticosteroids were not associated with COVID-19 infection or its severity.Allergic rhinitis protects individuals of all ages against COVID-19 infection [117].Thus the picture regarding the association between allergic rhinitis and COVID-19 is intriguing and not clearcut at the moment. Corticosteroid nasal spray is used to facilitate recovery of the sensation of smell lost in COVID-19 patients [118] and povidone nasal spray is used to decrease the load of COVID-19 in the nasopharxyn [119]. Intranasal anti-COVID-19 vaccines have been proposed [120]. Since SARS-CoV-2 coronavirus enters via the nasal passage a nasal spray like ARND would be an easy and convenient way of administration at home to prevent or treat COVID-19.

## Materials and methods

### ARND

Allergic Rhinitis Nose Drops (ARND, batch number 20010626) was provided by Lai Sing Medicine Factory Limited (Lai’s Medicine). It is composed of 11 Chinese medicinal herbs, including Centipedae Herba (*Pinyin: Ebushicao*, 23% by weight), Menthae Haplocalycis Herba (*Pinyin: Bohe*, 16%), Paeoniae Radix Alba (*Pinyin: Baishao*, 16%), Scutellariae Radix (*Pinyin: Huangqin*, 10%), Platycodonis Radix (*Pinyin: Jiegeng*, 6%), Glycyrrhizae Radix et Rhizoma (*Pinyin: Gancao*, 6%), Lonicerae Japonicae Flos (*Pinyin: Jinyinhua*, 5%), Saposhnikoviae Radix (*Pinyin: Fangfeng*, 5%), Jujubae Fructus (*Pinyin: Dazao*, 5%), Coptidis Rhizoma (*Pinyin: Huanglian*, 4%), and Citri Reticuulatae Pericarpium (*Pinyin: Chenpi*, 4%). ARND is a water extract of all the above herbal medicines. The yield of chemical components in ARND is calculated to be 33.2 ± 0.35 mg/mL (mean ± standard deviation, n = 3). The preparation of ARND is summarized in Additional file_METHOD_FIG_A1_TABLES_A1_12: ARND preparation.

### Cell culture

A549 lung cells (ATCC, Manassas, VA, USA; CCL-185), 293 T embryonic kidney cells (ATCC; CRL-3216), RAW264.7 macrophages (ATCC; TIB-71) and Calu-3 lung cells (Hunan Fenghui Biotechnology Co., Ltd, Hunan, PRC; CL0062) were maintained in complete medium of Dulbecco's Modified Eagle's Medium (DMEM; Gibco, Waltham, MA, USA), supplemented with 10% fetal bovine serum (FBS), and 1% antibiotic–antimycotic (Invitrogen, Waltham, MA, USA) in a humidified atmosphere of 5% CO_2_ at 37 °C. Cell subculture was performed at 60–70% confluence.

### Network pharmacology analysis

#### Predicted gene targets of ARND.

Network pharmacology analysis was performed in accordance with the study of Wang et al*.* [121]. Briefly, the chemical compounds of ARND were identified using the database Traditional Chinese Medicine Systems Pharmacology (TCMSP: https://tcmspw.com/tcmsp.php). Druggability analysis of the identified compounds in ARND was performed using Lipinski’s rule (LR) and referenced to the TCMSP database in terms of oral bioavailability (OB) and drug-likeness (DL), respectively. Data mining between identified compounds and gene targets was performed using String (https://string-db.org/) and Uniprot (http://beta.uniprot.org/) and Drug Bank (https://go.drugbank.com/).

#### Potential gene target of COVID-19

Data mining between disease and gene targets was performed with PubMed (https://pubmed.ncbi.nlm.nih.gov/), OMIM (https://www.omim.org/), GeneCard (https://www.genecards.org/) and String. ‘Corona Virus Disease 2019’ or ‘COVID-19’ was used as keyword searching and *Homo sapiens* was set for the species.

#### Network construction

Venn online software jvenn (http://jvenn.toulouse.inra.fr/) was used to illustrate the interaction between the drug-related targets of ARND and the targets of COVID-19. The targets intersected in both ARND and COVID-19 were submitted to CytoScape (https://cytoscape.org/index.html) to generate a drug–chemical-target network. Selected targets were further analyzed using STRING (https://string-db.org/) database to construct a protein–protein intersection (PPI) network model for identifying the key targets. The biological species was set at *Homo sapiens*, and the minimum intersection threshold was set at “high confidence” (> 0.4).

#### Enrichment analysis

Gene Ontology (GO) analysis and Kyoto Encyclopedia of Genes and Genomes (KEGG) pathway enrichment analysis were used to explore the biological pathways and potential functions. Selected targets with *p* < 0.01 were submitted to the Metascape platform (https://metascape.org/gp/index.html#/main/step1). The main biological processes and metabolic pathways were identified followed by enrichment analysis. OmicShare online tools (https://www.omicshare.com/) were used to visualize the data. A scheme of the workflow of network pharmacology analysis is shown in the graphical abstract.

### Pseudovirus infection assay

#### Generation of pseudovirus

To generate the pseudovirus for SARS-CoV-2, 293 T cells were seeded in a 100 mm TC-treated cell culture dish (SPL, Gyeonggi-do, South Korea) at 60–70% confluence. After 24 h, the DMEM + 10% FBS in the culture dish was replaced with 9 mL serum-free DMEM. Incubation of the cells was carried out at 37 °C for 1 h. For transfection, the transfection mix was prepared by mixing 6 μg of pVAX1-SARS-CoV-2-S (encoding Spike protein; NCBI accession number NC_045512) and 6 μg of pRGH (Red-Green-HIV-1 backbone) through PEI transfection reagent in the ratio of 1:3 in Opti-MEM medium (Gibco). The transfection mix was vortexed thoroughly before adding dropwise to the cells. Following incubation for 5–6 h, 6 mL complete medium was used to replace the medium, and the cells were subsequently incubated for 48 h. Transfection efficiency was evaluated under a fluorescence microscope (Additional file_METHOD_FIG_A1_TABLES_A1_12: Fig. A1). Medium was collected from the transfected cells, and centrifuged at 4000 rpm for 10 min at 4 °C. The supernatant containing the packaged pseudovirus was collected and filtered through a 0.45 μm filter, aliquoted and stored at − 80 °C until use.

#### Transient transfection of 293 T cells expressing ACE2 receptor

Plasmids encoding ACE2 (pcDNA3.1-ACE2-GFP, #154,962) were purchased from Addgene (Watertown, MA, USA). 293 T cell line was transiently transfected with expression plasmids encoding human ACE2 (293 T-ACE2 cells) at a 1.5:1 ratio using PEI reagent. Cells were used after 24 h post-transfection for seeding into 96-well plates for the infection assay on the next day.

#### Pseudovirus infection assay

A549 cells and 293 T-ACE2 cells were seeded in a 96-well plate for 24 h at a cell density of 7000 cells/well and 20,000 cells/well, respectively. Then the medium was replaced with 100 µL of complete medium containing ARND at concentrations ranging from 0.625 to 5 µL/mL and incubation was allowed to proceed for 2 h. The medium containing ARND was then discarded and replaced with the pseudovirus inoculum (in a total volume of 275 µL in each well) for infection to proceed for 2 h. The viral inoculum was then removed, and fresh complete medium (200 µL) was added to the cells and incubation was allowed to proceed for another 72 h.

After pseudovirus infection, the medium in a 96-well plate was removed and replaced with PBS for image acquisition under a 10 × objective using the Incucyte^®^ S3 instrument (Essen Bioscience, Ann Arbor, MI, USA). The images obtained were analyzed using the Incucyte S3 software to calculate the magnitude of the signals of fluorescence. The signal values were normalized by the uninfected cells and infected cells without ARND treatment.

#### Cell viability assay

A549 and 293 T-ACE2 cells were seeded at a cell density of 7 × 10^3^ cells/well and 2 × 10^4^ cells/well, respectively, in a 96-well plate for 24 h. Cells were treated with ARND at different concentrations for 24 h. To examine the viability of cells following ARND treatment, MTT (0.5 mg/mL) was added to the medium followed by incubation at 37 °C for 1 h. Formazan salts were dissolved in DMSO. The absorbance was determined at 570 nm with reference to 630 nm using a microplate reader (BioTek, Winooski, VT, USA).

### Assay of inhibition of spike protein-ACE2 interaction

Inhibition of binding between the receptor binding domain (RBD) of the SARS-CoV-2 or SARS-CoV-2 (B.1.617.2) Spike protein, namelySpike RBD or delta Spike RBD, and ACE2 receptor was studied using ELISA kits. Binding assay kits in the formats of ACE2 on plate (code: CoV-ACE2S2) and Spike RBD on plate (code: CoV-SACE2-1) were purchased from RayBiotech Life, Inc. (Peachtree Corners, GA, USA), whereas inhibitor screening kit for delta Spike RBD (catalog no.: EP-111) was purchased from ACROBiosystems (Newark, DE, USA). Different concentrations of ARND were used, and analysis was performed in accordance with the manufacturer’s protocol. The percentage of binding inhibition was computed as shown below.

$$\left[ {{1}{-}{\text{OD of test reagent well}}/{\text{OD of positive control}}} \right)] \times {1}00\%$$

### Treatment of RAW264.7 cells for use as a model of inflammation

RAW264.7 macrophages were seeded at a cell density of 3 × 10^4^ cells/cm^2^ in culture plates for 24 h. For the cell viability assay, cells were exposed to ARND at different concentrations for 48 h. For mRNA extraction, cells were exposed to different concentrations of ARND for 24 h and then co-treated with inflammatory M1 inducers (20 ng/mL IFN-γ and 100 ng/mL LPS) for a further 24 h. The cells were then harvested. Messenger RNA (mRNA) was then extracted from the samples for further analysis.

### Treatment of Calu-3 cells for use as a model of lung fibrosis

Calu-3 cells were seeded at a cell density of 1.2 × 10^4^ cells/cm^2^ in culture plates for 24 h. For the assay of viability of Calu-3 cells as a model of fibrosis, the cells were treated with different concentrations of ARND for 48 h or with H_2_O_2_ for 24 h. For mRNA extraction of fibrotic biomarkers, cells were treated with ARND at different concentrations for 24 h before treatment with 100 µM H_2_O_2_ to induce the fibrotic model for a further 24 h.

### Quantitative real-time PCR

Trizol (Invitrogen) was utilized to extract endogenous mRNA from cell samples. The mRNA was converted into complementary DNA using the commercial kit for reverse transcription in accordance with the manufacturer’s protocol. Expression levels of the inflammatory and fibrotic biomarkers were determined with real-time PCR using the respective primers at 50 nM concentration and β-actin was employed as the mRNA housekeeping control. The details of primer sequences used are shown in Additional file_METHOD_FIG_A1_TABLES_A1_12: Table A12.

### Statistical analysis

Data analysis was performed using one-way analysis of variance (ANOVA) and then Tukey's range test or Student's t-test. *p* < 0.05 was considered statistically significant. Comparisons between control and model group as well as model and different treatment groups were conducted. Data are presented as means ± standard error of the mean (SEM).

## Conclusion

In conclusion, the above in silico and in vitro results support that the proprietary Chinese medicine nasal spray ARND acts as an antiviral hygienic agent with a potential to prevent SARS-CoV-2 coronavirus infection by suppressing pseudovirus infection via targeting RBD (Delta)-ACE2 binding. ARND may be useful as a re-purposed herbal formulation nasal spray against COVID-19, which prompts further in vivo and clinical studies on ARND. The results provide support for further investigations on the effect of ARND in the live virus. Meanwhile, ARND also downregulated the levels of mRNA expression of proinflammatory transcription factors (NF-κB and c-Rel), proinflammatory cytokines (IL-1β, IL-6, TNF-α and CCL2) and NOS2 *in vitro*, which may be useful to attenuate the inflammatory response upon viral infection. Besides, ARND was able to downregulate mRNA expression levels of EMT (TGF-β, CXCL16, CCR6, IL-17, IL-25 and α-SMA) and ECM (Col-1a, Col-4a, elastin and Fn1), which may promote a better prognosis with a lowered risk of fibrosis in the lungs. All in all, ARND is devoid of toxicity and has potential of antiviral, antifibrotic and cytokine storm alleviating activities. ARND may offer hope in the current COVID-19 outbreak as well as in future pandemics of a similar nature. It remains to be seen whether ARND works well in the live virus and COVID-19 patients and can prevent recurrence of the viral disease.

## Supplementary Information

**Additional file 1. **A corresponding caption has been added in addition file accordingly. Captions of each additional fig and tables have been provided inside the manuscript accordingly. 

## Data Availability

Not applicable.

## Abbreviations

ARND: Allergic rhinitis nose drops
ACE2: Angiotensin converting enzyme 2
COVID-19: Coronavirus Disease 2019
PPI: Protein–protein interaction
GO: Gene ontology
KEGG: Kyoto encyclopedia of genes and genomes
ACE2: Angiotensin-converting enzyme 2
RBD: Receptor binding domain
DMEM: Dulbecco's Modified Eagle's Medium
FBS: Fetal bovine serum
mRNA: Messenger RNA
NFκB2: Nuclear factor kappa b subunit 2, c-Rel: REL proto-oncogene, NF-κB subunit
IL-1β: Interleukin 1 beta
IL-6: Interleukin 6
TNF-α: Tumor necrosis factor alpha
CCL2: C–C motif chemokine ligand 2
NOS2: Nitric oxide synthase 2
H_2_O_2_: Hydrogen peroxide
TGF-β: Transforming growth factor beta
CXCL16: Chemokine ligand 16
CXCR6: C-X-C chemokine receptor type 6
IL-17: Interleukin 17
IL-25: Interleukin 25
α-SMA: Alpha-smooth muscle actin
Col-1a: Collagen type I alpha
Col-4a: Collagen type IV alpha
Fn1: Fibronectin 1

## References

[1] World Health Organization Naming the coronavirus disease (COVID-19) and the virus that causes it. https://www.who.int/emergencies/diseases/novel-coronavirus-2019/technical-guidance/naming-the-coronavirus-disease-(covid-2019)-and-the-virus-that-causes-it (Accessed 17 Dec 2020).

[2] World Health Organization Q&As on COVID-19 and related health topics. https://www.who.int/emergencies/diseases/novel-coronavirus-2019/question-and-answers-hub (Accessed 16 May 2021).

[3] Tao Z, Liu J (2020). Immunologic features of the pathogenesis of COVID-19 (in Chinese). Chin J Microbiol Immunol.

[4] Fang W, Jiang J, Su L, Shu T, Liu H, Lai S, Ghiladi RA, Wang J (2021). The role of NO in COVID-19 and potential therapeutic strategies. Free Radic Biol Med.

[5] Jones SA, Hunter CA (2021). Is IL-6 a key cytokine target for therapy in COVID-19?. Nat Rev Immunol.

[6] Chua RL, Lukassen S, Trump S, Hennig BP, Wendisch D, Pott F, Debnath O, Thürmann L, Kurth F, Völker MT (2020). COVID-19 severity correlates with airway epithelium–immune cell interactions identified by single-cell analysis. Nat Biotechnol.

[7] Liu T, Zhang L, Joo D, Sun SC (2017). NF-κB signaling in inflammation. Signal Transduct Target Ther.

[8] Su CM, Wang L, Yoo D (2021). Activation of NF-κB and induction of proinflammatory cytokine expressions mediated by ORF7a protein of SARS-CoV-2. Sci Rep.

[9] Galea E, Feinstein DL (1999). Regulation of the expression of the inflammatory nitric oxide synthase (NOS2) by cyclic AMP. FASEB J.

[10] Guimarães LMF, Rossini CVT, Lameu C (2021). Implications of SARS-CoV-2 infection on eNOS and iNOS activity: consequences for the respiratory and vascular systems. Nitric Oxide.

[11] Diao B, Wang C, Tan Y, Chen X, Liu Y, Ning L, Chen L, Li M, Liu Y, Wang G (2020). Reduction and functional exhaustion of T Cells in patients with coronavirus disease 2019 (COVID-19). Front Immunol.

[12] Susanto AD, Triyoga PA, Isbaniah F, Fairuz A, Cendikiawan H, Zaron F, Aryanti I, Irbah SN, Hidayat M (2021). Lung Fibrosis Sequelae After Recovery from COVID-19 Infection. J Infect Dev Ctries.

[13] Ferreira-Gomes M, Kruglov A, Durek P, Heinrich F, Tizian C, Heinz GA, Pascual-Reguant A, Du W, Mothes R, Fan C (1961). SARS-CoV-2 in severe COVID-19 induces a TGF-β-dominated chronic immune response that does not target itself. Nat Commun.

[14] Xu J, Xu X, Jiang L, Dua K, Hansbro PM, Liu G (2020). SARS-CoV-2 induces transcriptional signatures in human lung epithelial cells that promote lung fibrosis. Respir Res.

[15] Stewart CA, Gay CM, Ramkumar K, Cargill KR, Cardnell RJ, Nilsson MB, Heeke S, Park EM, Kundu ST, Diao L (2021). Lung cancer models reveal severe acute respiratory syndrome coronavirus 2-induced epithelial-to-mesenchymal transition contributes to Coronavirus Disease 2019 pathophysiology. J Thorac Oncol.

[16] Di Gregorio J, Robuffo I, Spalletta S, Giambuzzi G, De Iuliis V, Toniato E, Martinotti S, Conti P, Flati V (2020). The epithelial-to-mesenchymal transition as a possible therapeutic target in fibrotic disorders. Front Cell Dev Biol.

[17] Stone RC, Pastar I, Ojeh N, Chen V, Liu S, Garzon KI, Tomic-Canic M (2016). Epithelial-mesenchymal transition in tissue repair and fibrosis. Cell Tissue Res.

[18] Ma Z, Yu R, Zhu Q, Sun L, Jian L, Wang X, Zhao J, Li C, Liu X (2020). CXCL16/CXCR6 axis promotes bleomycin-induced fibrotic process in MRC-5 cells via the PI3K/AKT/FOXO3a pathway. Int Immunopharmacol.

[19] Payne DJ, Dalal S, Leach R, Parker R, Griffin S, McKimmie CS, Cook GP, Richards SJ, Hillmen P, Munir T, Arnold L, et al. The CXCR6/CXCL16 axis links inflamm-aging to disease severity in COVID-19 patients. bioRxiv 2021.

[20] She YX, Yu QY, Tang XX (2021). Role of interleukins in the pathogenesis of pulmonary fibrosis. Cell Death Discov.

[21] Leask A, Abraham DJ (2004). TGF-β signaling and the fibrotic response. FASEB J.

[22] Wight TN, Potter-Perigo S (2011). The extracellular matrix: an active or passive player in fibrosis?. Am J Physiol Liver Physiol.

[23] Delpino MV, Quarleri J (2020). SARS-CoV-2 pathogenesis: Imbalance in the renin-angiotensin system favors lung fibrosis. Front Cell Infect Microbiol.

[24] Hussain S, Xie YJ, Li D, Malik SI, Hou JC, Leung ELH, Fan XX (2020). Current strategies against COVID-19. Chinese Med.

[25] Vahedi F, Lee AJ, Collins SE, Chew MV, Lusty E, Chen B, Dubey A, Richards CD, Feld JJ, Russell RS (2019). IL-15 and IFN-γ signal through the ERK pathway to inhibit HCV replication, independent of type I IFN signaling. Cytokine.

[26] Ryu G, Shin HW (2021). SARS-CoV-2 infection of airway epithelial cells. Immune Netw.

[27] Kim HS, Lee KE, Oh JH, Jung CS, Choi D, Kim Y, Jeon JS, Han DC, Noh H (2016). Cardiac arrest caused by nafamostat mesilate. Kidney Res Clin Pract.

[28] Fan Q, Zhang B, Ma J, Zhang S (2020). Safety profile of the antiviral drug remdesivir: an update. Biomed Pharmacother.

[29] Rai DK, Sharma P, Kumar R (2021). Post covid 19 pulmonary fibrosis. Is it real threat?. Indian J Tuberc.

[30] Hospital pharmacy professional committee of chinese pharmaceutical association expert consensus on rational drug use in clinical practice for COVID-19. Chinese J Hosp Pharm. 2020: 40; 593–605.

[31] National Health Commission of the People’s Republic of China Diagnosis and treatment of novel coronavirus pneumonia (Trial version 8). http://www.nhc.gov.cn/xcs/zhengcwj/202008/0a7bdf12bd4b46e5bd28ca7f9a7f5e5a.shtml (Accessed 17 Dec 17 2020).

[32] Ren J, Zhang AH, Wang XJ (2020). Traditional Chinese medicine for COVID-19 treatment. Pharmacol Res.

[33] Chan KW, Wong VT, Tang SCW (2020). COVID-19: An Update on the epidemiological, clinical, preventive and therapeutic evidence and guidelines of integrative Chinese-Western medicine for the management of 2019 novel coronavirus disease. Am J Chin Med.

[34] An X, Zhang Y, Duan L, Jin D, Zhao S, Zhou R, Duan Y, Lian F, Tong X (2021). The direct evidence and mechanism of traditional Chinese medicine treatment of COVID-19. Biomed Pharmacother.

[35] Xi S, Li Y, Yue L, Gong Y, Qian L, Liang T, Ye Y (2020). Role oftraditional Chinese Medicine in the management of viral pneumonia. Front Pharmacol.

[36] Dai YJ, Wan SY, Gong SS, Liu JC, Li F, Kou JP (2020). Recent advances of traditional Chinese medicine on the prevention and treatment of COVID-19. Chin J Nat Med.

[37] Chui SH, Shek SL, Fong MY, Szeto YT, Chan K (2010). A panel study to evaluate quality of life assessments in patients suffering from allergic rhinitis after treatment with a Chinese herbal nasal drop. Phyther Res.

[38] Hagemann J, Onorato GL, Jutel M, Akdis CA, Agache I, Zuberbier T, Czarlewski W, Mullol J, Bedbrook A, Bachert C (2021). Differentiation of COVID-19 signs and symptoms from allergic rhinitis and common cold: An ARIA-EAACI-GA 2 LEN consensus. Allergy.

[39] Manzo G (2020). COVID-19 as an immune complex hypersensitivity in antigen excess conditions: Theoretical pathogenetic process and suggestions for potentialtherapeutic interventions. Front Immunol.

[40] Zuo H, Lin YCD, Huang HY, Wang X, Tang Y, Hu Y, Kong X, Chen Q, Zhang Y, Hong HC (2021). The challenges and opportunities of traditional Chinese medicines against COVID-19: a way out from a network perspective. Acta Pharmacol Sin.

[41] Wu D, Hou X, Xia Z, Hao E, Xie J, Liang J, Liang Q, Du Z, Deng J (2021). Analysis on oral medication rules of traditional Chinese medicine prescriptions for prevention of COVID-19. Chin Herb Med.

[42] Luo H, Tang Q, Shang Y, Liang S, Yang M, Robinson N, Liu J (2020). Can Chinese medicine be used for prevention of corona virus disease 2019 (COVID-19)? A review of historical classics, research evidence and Ccurrent prevention programs. Chin J Integr Med.

[43] Yang KL, Gao Y, Yang FW, Liu M, Shi SZ, Chen YM, Zhang JH, Tian JH (2020). Analysis of traditional Chinese medicine from patent information sharing platform of coronavirus disease 2019 (COVID-19) (in Chinese). Zhongguo Zhong Yao Za Zhi.

[44] Hao E, Su Z, Gong Y, Du Z, Yang X, Huang C, Hou X, Deng J (2021). Analysis on application law of dampness-removing traditional Chinese medicines in treatment of coronavirus disease 2019. Chin Herb Med.

[45] Zhou Z, Gao N, Wang Y, Chang P, Tong Y, Fu S (2020). Clinical studies on the treatment of novel coronavirus pneumonia with traditional Chinese medicine — A literature analysis. Front Pharmacol.

[46] Shu Z, Zhou Y, Chang K, Liu J, Min X, Zhang Q, Sun J, Xiong Y, Zou Q, Zheng Q (2020). Clinical features and the traditional Chinese medicine therapeutic characteristics of 293 COVID-19 inpatient cases. Front Med.

[47] Wang C, Ming H, Jia W, Su W, Zhan LR, Luo D, Yang JY (2020). Analysis of medication regularity and pharmacodynamic characteristics of traditional Chinese medicine treatment in 444 severe cases of COVID-19 (in Chinese). Zhongguo Zhong Yao Za Zhi.

[48] Ang L, Lee HW, Kim A, Lee MS (2020). Herbal medicine for the management of COVID-19 during the medical observation period: a review of guidelines. Integr Med Res.

[49] Niu W, Wu F, Cui H, Cao W, Chao Y, Wu Z, Fan M, Liang C (2020). Network pharmacology analysis to identify phytochemicals in traditional Chinese medicines that may regulate ACE2 for the treatment of COVID-19. Evid Based Complement Altern Med..

[50] Zhou P, Yang XL, Wang XG, Hu B, Zhang L, Zhang W, Si HR, Zhu Y, Li B, Huang CL (2020). A pneumonia outbreak associated with a new coronavirus of probable bat origin. Nature.

[51] Mody V, Ho J, Wills S, Mawri A, Lawson L, Ebert MC, Fortin GM, Rayalam S, Taval S (2021). Identification of 3-chymotrypsin like protease (3CLPro) inhibitors as potential anti-SARS-CoV-2 agents. Commun. Biol..

[52] Ma J, Huo X-Q, Chen X, Zhu W-X, Yao M-C, Qiao Y-J, Zhang Y-L (2020). Study on screening potential traditional Chinese medicines against 2019-nCoV based on Mpro and PLP (in Chinese). Zhongguo Zhong Yao Za Zhi.

[53] Zeng C, Yuan Z, Pan X, Zhang J, Zhu J, Zhou F, Shan Z, Yuan Y, Ye R, Cheng J (2020). Efficacy of traditional Chinese medicine, Maxingshigan-Weijing in the management of COVID-19 patients with severe acute respiratory syndrome: a structured summary of a study protocol for a randomized controlled trial. Trials.

[54] Yu XQ, Xue CC, Zhou ZW, Li CG, Du YM, Liang J, Zhou SF (2008). *In vitro* and *in vivo* neuroprotective effect and mechanisms of glabridin, a major active isoflavan from *Glycyrrhiza glabra* (licorice). Life Sci.

[55] Gao J, Ding Y, Wang Y, Liang P, Zhang L, Liu R (2021). Oroxylin A is a severe acute respiratory syndrome coronavirus 2-spiked pseudotyped virus blocker obtained from Radix Scutellariae using angiotensin-converting enzyme II/cell membrane chromatography. Phyther Res.

[56] Bai LL, Chen H, Zhou P, Yu J (2021). Identification of tumor necrosis factor-alpha (TNF-α) inhibitor in rheumatoid arthritis using network pharmacology and molecular docking. Front Pharmacol.

[57] Narkhede RR, Pise AV, Cheke RS, Shinde SD (2020). Recognition of natural products as potential inhibitors of COVID-19 main protease (Mpro): *In-silico* evidences. Nat Products Bioprospect.

[58] Li CL, Tan LH, Wang YF, Luo CD, Chen HB, Lu Q, Li YC, Yang XB, Chen JN, Liu YH, Xie JH (2019). Comparison of anti-inflammatory effects of berberine, and its natural oxidative and reduced derivatives from Rhizoma Coptidis in vitro and in vivo. Phytomedicine.

[59] Wang Z, Li K, Maskey AR, Huang W, Toutov AA, Yang N, Srivastava K, Geliebter J, Tiwari R, Miao M (2021). A small molecule compound berberine as an orally active therapeutic candidate against COVID-19 and SARS: A computational and mechanistic study. FASEB J.

[60] Khan S, Siddiqui F (2020). Beta-sitosterol: as immunostimulant, antioxidant and inhibitor of SARS-CoV-2 spike glycoprotein. Arch Pharmacol Ther.

[61] Zhao H, Zeng S, Chen L, Sun Q, Liu M, Yang H, Ren S, Ming T, Meng X, Xu H (2021). Updated pharmacological effects of Lonicerae japonicae flos, with a focus on its potential efficacy on coronavirus disease-2019 (COVID-19). Curr Opin Pharmacol.

[62] Liu H, Ye F, Sun Q, Liang H, Li C, Li S, Lu R, Huang B, Tan W, Lai L (2021). Scutellaria baicalensis extract and baicalein inhibit replication of SARS-CoV-2 and its 3C-like protease in vitro. J Enzyme Inhib Med Chem.

[63] Udrea A-M, Mernea M, Buiu C, Avram S (2020). *Scutellaria baicalensis* flavones as potent drugs against acute respiratory injury during SARS-CoV-2 infection: Structural biology approaches. Processes.

[64] Zheng Y, Zeng X, Chen P, Chen T, Peng W, Su W (2020). Integrating pharmacology and gut microbiota analysis to explore the m of Citri Reticulatae Pericarpium against reserpine-induced spleen deficiency in rats. Front Pharmacol.

[65] Huang Y-F, Bai C, He F, Xie Y, Zhou H (2020). Review on the potential action mechanisms of Chinese medicines in treating coronavirus disease 2019 (COVID-19). Pharmacol Res.

[66] Rehman MT, AlAjmi MF, Hussain A (2021). Natural compounds as inhibitors of SARS-CoV-2 main protease (3CLpro): A molecular docking and simulation approach to combat COVID-19. Curr Pharm Des.

[67] Yu R, Chen L, Lan R, Shen R, Li P (2020). Computational screening of antagonists against the SARS-CoV-2 (COVID-19) coronavirus by molecular docking. Int J Antimicrob Agents.

[68] Derosa G, Maffioli P, D’Angelo A, Di Pierro F (2021). A role for quercetin in coronavirus disease 2019 (COVID-19). Phyther Res.

[69] Patočka J, Navrátilová Z, Kuča K, Olekšák P, Kumar Killi U (2021). Can baicalein become a new drug for COVID-19?. Mil Med Sci Lett.

[70] Pooja M, Reddy GJ, Hema K, Dodoala S, Koganti B (2021). Unravelling high-affinity binding compounds towards transmembrane protease serine 2 enzyme in treating SARS-CoV-2 infection using molecular modelling and docking studies. Eur J Pharmacol.

[71] Xia J, Kotani A, Hakamata H, Kusu F (2006). Determination of hesperidin in Pericarpium Citri Reticulatae by semi-micro HPLC with electrochemical detection. J Pharm Biomed Anal.

[72] Haggag YA, El-Ashmawy NE, Okasha KM (2020). Is hesperidin essential for prophylaxis and treatment of COVID-19 Infection?. Med Hypotheses.

[73] Bailly C, Vergoten G (2020). Glycyrrhizin: An alternative drug for the treatment of COVID-19 infection and the associated respiratory syndrome?. Pharmacol Ther.

[74] Gomaa AA, Abdel-Wadood YA (2021). The potential of glycyrrhizin and licorice extract in combating COVID-19 and associated conditions. Phytomed Plus.

[75] Ding H, Deng W, Ding L, Ye X, Yin S, Huang W (2020). Glycyrrhetinic acid and its derivatives as potential alternative medicine to relieve symptoms in nonhospitalized COVID-19 patients. J Med Virol.

[76] Gao QH, Wu CS, Yu JG, Wang M, Ma YJ, Li CL (2012). Textural characteristic, antioxidant activity, sugar, organic acid, and phenolic profiles of 10 promising jujube (*Ziziphus jujuba* Mill.) selections. J Food Sci.

[77] Sun Y, Ding S, Huang H, Hu Y (2017). Ionic liquid-based enzyme-assisted extraction of chlorogenic acid from Flos Lonicera Japonicae. Bioresour Bioprocess.

[78] Elfiky AA (2021). Natural products may interfere with SARS-CoV-2 attachment to the host cell. J Biomol Struct Dyn.

[79] Alhadrami HA, Sayed AM, Sharif AM, Azhar EI, Rateb ME (2021). Olive-derived triterpenes suppress SARS COV-2 main protease: a promising scaffold for future therapeutics. Molecules.

[80] Son J, Lee SY (2020). Therapeutic potential of ursonic acid: Comparison with ursolic acid. Biomolecules.

[81] Kumar A, Choudhir G, Shukla SK, Sharma M, Tyagi P, Bhushan A, Rathore M (2021). Identification of phytochemical inhibitors against main protease of COVID-19 using molecular modeling approaches. J Biomol Struct Dyn.

[82] Lin C, Tsai F, Hsue Y, Hof T, Wang G, Chiu Y, Ham H, Yang J (2021). Study of baicalin toward COVID-19 treatment: *In silico* target analysis and *in vitro* inhibitory effects on SARS-CoV-2 proteases. Biomed Hub.

[83] Kim TY, Jeon S, Jang Y, Gotina L, Won J, Ju YH, Kim S, Jang MW, Won W, Park MG (2021). Platycodin D, a natural component of *Platycodon grandiflorum*, prevents both lysosome- and TMPRSS2-driven SARS-CoV-2 infection by hindering membrane fusion. Exp Mol Med.

[84] Makowski L, Olson-Sidford W, Weisel JW (2021). Biological and clinical consequences of integrin binding via a rogue RGD motif in the SARS CoV-2 spike protein. Viruses.

[85] Petruk G, Puthia M, Petrlova J, Samsudin F, Strömdahl AC, Cerps S, Uller L, Kjellström S, Bond PJ, Schmidtchen A (2020). SARS-CoV-2 spike protein binds to bacterial lipopolysaccharide and boosts proinflammatory activity. J Mol Cell Biol.

[86] Ripa I, Andreu S, López-Guerrero JA, Bello-Morales R (2021). Membrane rafts: portals for viral entry. Front Microbiol.

[87] Sellegounder D, Zafari P, Rajabinejad M, Taghadosi M, Kapahi P (2021). Advanced glycation end products (AGEs) and its receptor, RAGE, modulate age-dependent COVID-19 morbidity and mortality. A review and hypothesis. Int Immunopharmacol.

[88] Schmidt F, Weisblum Y, Muecksch F, Hoffmann H-H, Michailidis E, Lorenzi JCC, Mendoza P, Rutkowska M, Bednarski E, Gaebler C (2020). Measuring SARS-CoV-2 neutralizing antibody activity using pseudotyped and chimeric viruses. J Exp Med.

[89] Neerukonda SN, Vassell R, Herrup R, Liu S, Wang T, Takeda K, Yang Y, Lin TL, Wang W, Weiss CD (2021). Establishment of a well-characterized SARSCoV-2 lentiviral pseudovirus neutralization assay using 293T cells with stable expression of ACE2 and TMPRSS2. PLoS ONE.

[90] Lujan H, Criscitiello MF, Hering AS, Sayes CM (2019). Refining *in vitro* toxicity models: comparing baseline characteristics of lung cell types. Toxicol Sci.

[91] Lei C, Qian K, Li T, Zhang S, Fu W, Ding M, Hu S (2020). Neutralization of SARS-CoV-2 spike pseudotyped virus by recombinant ACE2-Ig. Nat Commun.

[92] Liu CJ (2020). Possibility of SARS-CoV-2 infection and transmission through the digestive tract. World Chinese J Dig.

[93] Yu S, Zhu Y, Xu J, Yao G, Zhang P, Wang M, Zhao Y, Lin G, Chen H, Chen L (2021). Glycyrrhizic acid exerts inhibitory activity against the spike protein of SARS-CoV-2. Phytomedicine.

[94] Chen H, Du Q. Potential natural compounds for preventing SARS-CoV-2 (2019-nCoV) infection. Preprints 2020, 2020010358.

[95] Centers for Disease Control and Prevention Emerging SARS-CoV-2 Variants. https://www.cdc.gov/coronavirus/2019-ncov/science/science-briefs/scientific-brief-emerging-variants.html (Accessed 25 Jul 2021). 34009774

[96] World Health Organization Tracking SARS-CoV-2 variants. https://www.who.int/en/activities/tracking-SARS-CoV-2-variants/ (Accessed 25 Jul 2021).

[97] Antony P, Vijayan R (2021). Role of SARS-CoV-2 and ACE2 variations in COVID-19. Biomed J.

[98] Stancioiu F, Papadakis G, Kteniadakis S, Izotov B, Coleman M, Spandidos D, Tsatsakis A (2020). A dissection of SARS-CoV2 with clinical implications (Review). Int J Mol Med.

[99] Fiege JK, Thiede JM, Nanda HA, Matchett WE, Moore PJ, Montanari NR, Thielen BK, Daniel J, Stanley E, Hunter RC (2021). Single cell resolution of SARS-CoV-2 tropism, antiviral responses, and susceptibility to therapies in primary human airway epithelium. PLOS Pathog.

[100] Guan F, Lam W, Hu R, Kim YK, Han H, Cheng Y-C (2018). Majority of Chinese medicine herb category “Qing Re Yao” have multiple mechanisms of anti-inflammatory activity. Sci Rep.

[101] Bost P, Giladi A, Liu Y, Bendjelal Y, Xu G, David E, Blecher-Gonen R, Cohen M, Medaglia C, Li H (2020). Host-viral infection maps reveal signatures of severe COVID-19 patients. Cell.

[102] Liao M, Liu Y, Yuan J, Wen Y, Xu G, Zhao J, Cheng L, Li J, Wang X, Wang F (2020). Single-cell landscape of bronchoalveolar immune cells in patients with COVID-19. Nat Med.

[103] Chernyak BV, Popova EN, Prikhodko AS, Grebenchikov OA, Zinovkina LA, Zinovkin RA (2020). COVID-19 and Oxidative Stress. Biochem.

[104] Cheresh P, Kim S-J, Tulasiram S, Kamp DW (2013). Oxidative stress and pulmonary fibrosis. Biochim Biophys Acta Mol Basis Dis.

[105] Fujita T, Maruyama M, Araya J, Sassa K, Kawagishi Y, Hayashi R, Matsui S, Kashii T, Yamashita N, Sugiyama E (2002). Hydrogen peroxide induces upregulation of Fas in human airway epithelial cells via the activation of PARP-p53pathway. Am J Respir Cell Mol Biol.

[106] Song X, Bai S, He N, Wang R, Xing Y, Lv C, Yu F (2021). Real-time evaluation of hydrogen peroxide injuries in pulmonary fibrosis mice models with a mitochondria-targeted near-infrared fluorescent probe. ACS Sensors.

[107] Li L, Kan L (2017). Traditional Chinese medicine for pulmonary fibrosis therapy: progress and future prospects. J Ethnopharmacol.

[108] Di Pierro F, Iqtadar S, Khan A, Ullah Mumtaz S, Masud Chaudhry M, Bertuccioli A, Derosa G, Maffioli P, Togni S, Riva A (2021). Potential clinical benefits of quercetin in the early stage of COVID-19: Results of a second, pilot, randomized, controlled and open-label clinical trial. Int J Gen Med.

[109] Ventura MT, Piccinni T, Matino MG, Giuliano G, Corato R, Napoli Di, Tursi A (2001). Retrospective study on flutiocasone propionate aqueous nasal spray efficacy in patients with allergic rhinitis:evaluation of clinical and laboratory parameters. Allergy.

[110] Leung KF, Liu FB, Zhao L, Fang JQ, Chan K, Lin LZ (2005). Development and validation of the Chinese quality of life instrument. Health Qual Life Outcomes.

[111] Hung IF, Lung KC, Tso EY, Liu R, Chung TW, Chu MY, Ng YY, Lo J, Chan J, Tam AR, Shum HP, Chan V, Wu AK, Sin KM, Leung WS, Law WL, Lung DC, Sin S, Yeung P, Yip CC, Zhang RR, Fung AY, Yan EY, Leung KH, Ip JD, Chu AW, Chan WM, Ng AC, Lee R, Fung K, Yeung A, Wu TC, Chan JW, Yan WW, Chan WM, Chan JF, Lie AK, Tsang OT, Cheng VC, Que TL, Lau CS, Chan KH, To KK, Yuen KY (2020). Triple combination of interferon beta-1b, lopinavir-ritonavir, and ribavirin in the treatment of patients admitted to hospital with COVID-19: an open-label, randomised, phase 2 trial. Lancet.

[112] Hazan S, Dave S, Gunaratne AW, Dolai S, Clancy RL, McCullough PA, Borody TJ (2022). Effectiveness of ivermectin-based multidrug therapy in severely hypoxic, ambulatory COVID-19 patients. Future Microbiol.

[113] Yang JM, Koh HY, Moon SY, Yoo IK, Ha EK, You S, Kim SY, Yon DK, Lee SW (2020). Allergic disorders and susceptibility to and severity of COVID-19: a nationwide cohort study. J Allergy Clin Immunol.

[114] Guvey A (2021). How does allergic rhinitis impact the severity of COVID-19?: a case-control study. Eur Arch Otorhinolaryngol.

[115] Gani F, Cottini M, Landi M, Berti A, Comberiati P, Peroni D, Senna G, Lombardi C (2022). Allergic rhinitis and COVID-19: friends or foes?. Eur Ann Allergy Clin Immunol.

[116] Vezir E, Hizal M, Cura Yayla B, Aykac K, Yilmaz A, Kaya G, Oygar PD, Ozsurekci Y, Ceyhan M (2021). Does aeroallergen sensitivity and allergic rhinitis in children cause milder COVID-19 infection?. Allergy Asthma Proc.

[117] Ren J, Pang W, Luo Y, Cheng D, Qiu K, Rao Y, Zheng Y, Dong Y, Peng J, Hu Y, Ying Z, Yu H, Zeng X, Zong Z, Liu G, Wang D, Wang G, Zhang W, Xu W, Zhao Y (2022). Impact of allergic rhinitis and asthma on COVID-19 infection, hospitalization, and mortality. J Allergy Clin Immunol Pract.

[118] Abdelalim AA, Mohamady AA, Elsayed RA, Elawady MA, Ghallab AF (2021). Corticosteroid nasal spray for recovery of smell sensation in COVID-19 patients: a randomized controlled trial. Am J Otolaryngol.

[119] Guenezan J, Garcia M, Strasters D, Jousselin C, Lévêque N, Frasca D, Mimoz O (2021). Povidone iodine mouthwash, gargle, and nasal spray to reduce nasopharyngeal viral load in patients with COVID-19: a randomized clinical trial. JAMA Otolaryngol Head Neck Surg.

[120] Chavda VP, Vora LK, Pandya AK, Patravale VB (2021). Intranasal vaccines for SARS-CoV-2: From challenges to potential in COVID-19 management. Drug Discov Today.

[121] Wang S, Tong Y, Ng TB, Lao L, Lam JKW, Zhang KY, Zhang ZJ, Sze SCW (2015). Network pharmacological identification of active compounds and potential actions of Erxian decoction in alleviating menopause-related symptoms. Chin Med.

