# Supplementary material for: An anti-inflammatory and anti-fibrotic proprietary Chinese medicine nasal spray designated as Allergic Rhinitis Nose Drops (ARND) with potential to prevent SARS-CoV-2 coronavirus infection by targeting RBD (Delta)- angiotensin converting enzyme 2 (ACE2) binding
Source: Chin Med. 2022 Jul 27;17:88. doi: 10.1186/s13020-022-00635-2 (PMC9328017; doi:10.1186/s13020-022-00635-2)
Supplement: Supplementary file 1 — Additional file 1. A corresponding caption has been added in addition file accordingly. Captions of each additional fig and tables have been provided inside the manuscript accordingly. [file 13020_2022_635_MOESM1_ESM.pdf]

Additional information: Contents

1 Additional materials and methods **2**

1.1 ARND preparation 2

2 Additional figures **3**

Figure A1 Co-transfected 293T cells with RGH and pVAX1-SARS-CoV-2-S showed both red and green fluorescence when merged with brightfield. White arrows indicate transfected cells. 3

3 Additional tables **4**

Table A1 Results of intersection analysis. 4

Table A2 List of gene targets of ARND overlapping with COVID-19. 4

Table A3 List of compounds in ARND had gene targets overlapping with COVID-19. 5

Table A4 Target-compound-drugs list of 52 identified compounds and 118 identified gene targets of ARND exerting its effects on the coronavirus diseases. The herbs are numbered as follows. 1 = Centipedae Herba, 2 = Coptidis Rhizoma, 3 = Lonicerae Japonicae Flos, 4 = Scutellariae Radix, 5 = Menthae Haplocalycis Herba, 6 = Platycodonis Radix, 7 = Saposhnikoviae Radix, 8 = Citri Reticuulatae Pericarpium, 9 = Paeoniae Radix Alba, 10 = Glycyrrhizae Radix et Rhizoma, and 11 = Jujubae Fructus. 5

Table A5 List of gene targets in PPI network with average number of neighbors greater than 43.864. 16

Table A6 List of top 10 site of expression 17

Table A7 List of top 10 biological pathways. 17

Table A8 List of top 20 GO terms of biological process enrichment analysis. 18

Table A9 List of top 20 GO terms of cellular component enrichment analysis. 19

Table A10 List of top 20 GO terms of molecular function enrichment analysis. 20

Table A11 List of top 20 KEGG signaling pathways of enrichment analysis. 21

Table A12 Primer sequences of genes used for mouse RAW264.7 cells and human Calu-3 cells in quantitative real-time PCR analysis. 22

1 Additional materials and methods

1.1 ARND preparation

The brief preparation of ARND was provided by Lai Sing Medicine Factory Limited (Lai's Medicine). To prepare ARND, the required herbal medicines were firstly washed in a water tank. The cleaned herbs were then steamed for 1-2 hrs to remove impurities. After that, the herbs were oven-dried under 60 – 70 ^{o}C for 3-5 hrs. The dried herbs were placed in a stainless-steel container with double steamed rice wine followed by heating under 80 ^{o}C for 8 hrs. Subsequently, the mixture was filtered, and the obtained filtrate was loaded into bottles for ARND product.

2 Additional figures

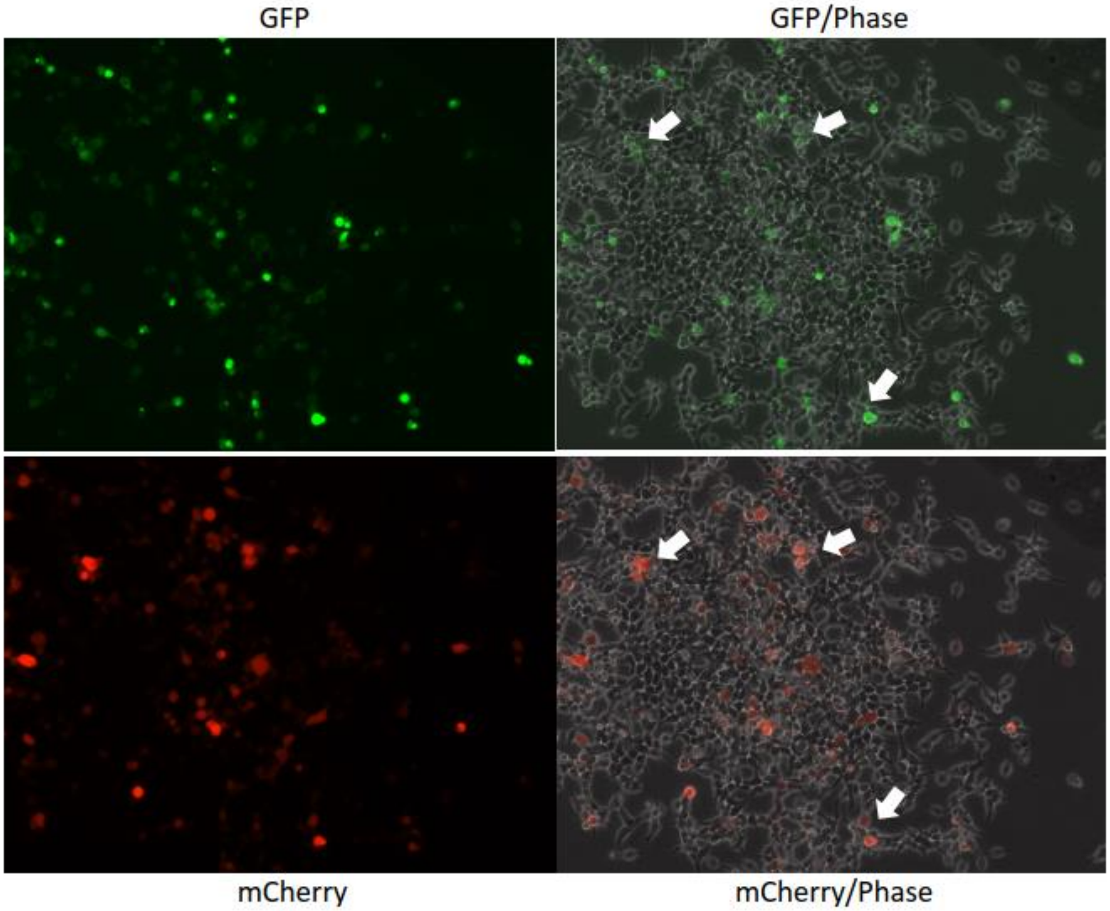

Figure A1 Co-transfected 293T cells with RGH and pVAX1-SARS-CoV-2-S showed both red and green fluorescence when merged with brightfield. White arrows indicate transfected cells.

3 Additional tables

Table A1 Results of intersection analysis.

Code	Deseription	Drugs	Chemicals	Targets
ARND	ARND	11	229	554
ARNDX03	COVID19			805
ARNDA04	ARND-COVID19	11	52	118

Table A2 List of gene targets of ARND overlapping with COVID-19.

NOS2	SNCA	CSNK2A1	CACNA1S	CYP3A4	ESR1	MAPK8
BAK1	NR3C2	CYP1B1	NOS3	SCN5A	VEGFA	SRC
UGT1A1	HMOX1	EZH2	SIRT3	CYCS	TMPRSS11D	CXCL10
HSD11B2	EFNA5	BDNF	CYP2C8	C3	MYC	TYR
CYP2D6	SOD1	MPO	PKM	IL5	IL13	CCL5
APOE	IL1RN	SCARB1	GCG	CRP	STAT1	TP53
AGT	SEC14L2	COL3A1	LCN2	CTSB	INS	IL1B
NFE2L2	ODC1	CYP1A2	BECN1	TNF	CSF2	CDK4
UGT1A8	CDKN1A	NQO1	NOTCH1	EGF	SIRT1	TGFB1
SPP1	ATG12	TOP2A	BAX	IL1A	CFTR	MME
CASP1	IL10	NFKBIA	CCL2	ICAM1	ADIPOQ	GSK3B
TLR5	MAPK1	ALB	IL6	CAV1	TBK1	ITGAM
CCL4	EGFR	TLR2	CASP3	BCL2L1	DPP4	TTR
CXCL8	PLAT	IL2	MCL1	MAPK3	JUN	F3
IL17A	ERBB2	IFNG	CA2	TLR4	CD40LG	TOP1
MAPK14	KCNH2	HSPA5	SERPINE1	AKT1	CCL3	PTGS2
STAT3	PPARG	XPO1	DUSP1	BACE1	MTOR	

Table A3 List of compounds in ARND had gene targets overlapping with COVID-19.

Luteolin	Stigmasterol	Acacetin	Naringenin
Ent-epicatechin	Aloe-emodin	Paeoniflorin	Ruvoside_qt
(-)-catechin	(+)-catechin	Ammidin	Cis-dihydroquercetin
Quercetin	Licochalcone a	Isoimperatorin	Licochalcone b
Wogonin	Vestitol	Phytofluene	Glabridin
Mairin	Stepholidine	Baicalein	Eriodictyol
Isorhamnetin	Palmatine	Beta-carotene	Genkwanin
Beta-sitosterol	Fumarine	Diosmetin	Nobiletin
Sitosterol	Berberine	Oroxylin a	Nuciferin
Formononetin	Coptisine	Panicolin	Helenalin
Calycosin	Supraene	Zinc03978781	Stepharine
Kaempferol	(s)-coclaurine	Chryseriol	Zizyphus saponin i_qt
Coumestrol	Jujubasaponin v_qt	Mauritine d	Obacunone

Table A4 Target-compound-drugs list of 52 identified compounds and 118 identified gene targets of ARND exerting its effects on the coronavirus diseases. The herbs are numbered as follows. 1 = Centipedae Herba, 2 = Coptidis Rhizoma, 3 = Lonicerae Japonicae Flos, 4 = Scutellariae Radix, 5 = Menthae Haplocalycis Herba, 6 = Platycodonis Radix, 7 = Saposhnikoviae Radix, 8 = Citri Reticuulatae Pericarpium, 9 = Paeoniae Radix Alba, 10 = Glycyrrhizae Radix et Rhizoma, and 11 = Jujubae Fructus.

Mol ID	Chemical name	Target	Drug(s)		
MOL000006	Luteolin	AGT	3	5	6
MOL000006	Luteolin	AKT1	3	5	6
MOL000006	Luteolin	BCL2L1	3	5	6
MOL000006	Luteolin	C3	3	5	6
MOL000006	Luteolin	CASP3	3	5	6
MOL000006	Luteolin	CDK4	3	5	6
MOL000006	Luteolin	CSNK2A1	3	5	6
MOL000006	Luteolin	CYP1B1	3	5	6
MOL000006	Luteolin	EGFR	3	5	6
MOL000006	Luteolin	ERBB2	3	5	6
MOL000006	Luteolin	EZH2	3	5	6

MOL000006	Luteolin	HMOX1	3	5	6	
MOL000006	Luteolin	IL1B	3	5	6	
MOL000006	Luteolin	JUN	3	5	6	
MOL000006	Luteolin	LCN2	3	5	6	
MOL000006	Luteolin	MAPK1	3	5	6	
MOL000006	Luteolin	MAPK3	3	5	6	
MOL000006	Luteolin	MAPK8	3	5	6	
MOL000006	Luteolin	MTOR	3	5	6	
MOL000006	Luteolin	NFE2L2	3	5	6	
MOL000006	Luteolin	NOS2	3	5	6	
MOL000006	Luteolin	PKM	3	5	6	
MOL000006	Luteolin	PPARG	3	5	6	
MOL000006	Luteolin	STAT3	3	5	6	
MOL000006	Luteolin	TBK1	3	5	6	
MOL000006	Luteolin	TLR4	3	5	6	
MOL000006	Luteolin	TLR5	3	5	6	
MOL000006	Luteolin	TOP1	3	5	6	
MOL000006	Luteolin	TP53	3	5	6	
MOL000006	Luteolin	TTR	3	5	6	
MOL000006	Luteolin	VEGFA	3	5	6	
MOL000073	Ent-Epicatechin	BACE1	4			
MOL000073	Ent-Epicatechin	CSF2	4			
MOL000073	Ent-Epicatechin	HMOX1	4			
MOL000073	Ent-Epicatechin	IL6	4			
MOL000073	Ent-Epicatechin	NOS2	4			
MOL000073	Ent-Epicatechin	NOS3	4			
MOL000073	Ent-Epicatechin	PTGS2	4			
MOL000096	(-)-Catechin	ESR1	11			
MOL000096	(-)-Catechin	PTGS2	11			
MOL000096	(-)-Catechin	PPARG	11			
MOL000098	Quercetin	AKT1	1	3	10	11
MOL000098	Quercetin	CASP3	1	3	10	11
MOL000098	Quercetin	CCL2	1	3	10	11
MOL000098	Quercetin	CSF2	1	3	10	11
MOL000098	Quercetin	CYP1A2	1	3	10	11
MOL000098	Quercetin	CYP1B1	1	3	10	11

MOL000098	Quercetin	CYP2C8	1	3	10	11
MOL000098	Quercetin	CYP3A4	1	3	10	11
MOL000098	Quercetin	HMOX1	1	3	10	11
MOL000098	Quercetin	IL6	1	3	10	11
MOL000098	Quercetin	JUN	1	3	10	11
MOL000098	Quercetin	MAPK8	1	3	10	11
MOL000098	Quercetin	MCL1	1	3	10	11
MOL000098	Quercetin	PTGS2	1	3	10	11
MOL000098	Quercetin	SIRT1	1	3	10	11
MOL000098	Quercetin	SRC	1	3	10	11
MOL000098	Quercetin	TP53	1	3	10	11
MOL000098	Quercetin	UGT1A1	1	3	10	11
MOL000098	Quercetin	UGT1A8	1	3	10	11
MOL000098	Quercetin	VEGFA	1	3	10	11
MOL000098	Quercetin	PPARG	1	3	10	11
MOL000098	Quercetin	PTGS2	1	3	10	11
MOL000098	Quercetin	DPP4	1	3	10	11
MOL000098	Quercetin	TOP2A	1	3	10	11
MOL000098	Quercetin	KCNH2	1	3	10	11
MOL000098	Quercetin	SCN5A	1	3	10	11
MOL000098	Quercetin	NOS3	1	3	10	11
MOL000098	Quercetin	EGFR	1	3	10	11
MOL000098	Quercetin	AKT1	1	3	10	11
MOL000098	Quercetin	VEGFA	1	3	10	11
MOL000098	Quercetin	BCL2L1	1	3	10	11
MOL000098	Quercetin	CDKN1A	1	3	10	11
MOL000098	Quercetin	BAX	1	3	10	11
MOL000098	Quercetin	MAPK1	1	3	10	11
MOL000098	Quercetin	IL10	1	3	10	11
MOL000098	Quercetin	EGF	1	3	10	11
MOL000098	Quercetin	TNF	1	3	10	11
MOL000098	Quercetin	JUN	1	3	10	11
MOL000098	Quercetin	IL6	1	3	10	11
MOL000098	Quercetin	CASP3	1	3	10	11
MOL000098	Quercetin	TP53	1	3	10	11
MOL000098	Quercetin	NFKBIA	1	3	10	11

MOL000098	Quercetin	ODC1	1	3	10	11
MOL000098	Quercetin	TOP1	1	3	10	11
MOL000098	Quercetin	SOD1	1	3	10	11
MOL000098	Quercetin	HSPA5	1	3	10	11
MOL000098	Quercetin	ERBB2	1	3	10	11
MOL000098	Quercetin	PPARG	1	3	10	11
MOL000098	Quercetin	HMOX1	1	3	10	11
MOL000098	Quercetin	CYP3A4	1	3	10	11
MOL000098	Quercetin	CYP1A2	1	3	10	11
MOL000098	Quercetin	CAV1	1	3	10	11
MOL000098	Quercetin	MYC	1	3	10	11
MOL000098	Quercetin	F3	1	3	10	11
MOL000098	Quercetin	ICAM1	1	3	10	11
MOL000098	Quercetin	IL1B	1	3	10	11
MOL000098	Quercetin	CCL2	1	3	10	11
MOL000098	Quercetin	CXCL8	1	3	10	11
MOL000098	Quercetin	NOS3	1	3	10	11
MOL000098	Quercetin	TGFB1	1	3	10	11
MOL000098	Quercetin	IL2	1	3	10	11
MOL000098	Quercetin	CYP1B1	1	3	10	11
MOL000098	Quercetin	PLAT	1	3	10	11
MOL000098	Quercetin	SERPINE1	1	3	10	11
MOL000098	Quercetin	IFNG	1	3	10	11
MOL000098	Quercetin	IL1A	1	3	10	11
MOL000098	Quercetin	MPO	1	3	10	11
MOL000098	Quercetin	TOP2A	1	3	10	11
MOL000098	Quercetin	NFE2L2	1	3	10	11
MOL000098	Quercetin	NQO1	1	3	10	11
MOL000098	Quercetin	COL3A1	1	3	10	11
MOL000098	Quercetin	CRP	1	3	10	11
MOL000098	Quercetin	CXCL10	1	3	10	11
MOL000098	Quercetin	SPP1	1	3	10	11
MOL000098	Quercetin	CD40LG	1	3	10	11
MOL000173	Wogonin	BAK1	4	7		
MOL000173	Wogonin	BCL2L1	4	7		
MOL000173	Wogonin	CASP3	4	7		

MOL000173	Wogonin	CCL2	4	7				
MOL000173	Wogonin	HMOX1	4	7				
MOL000173	Wogonin	MAPK1	4	7				
MOL000173	Wogonin	MAPK3	4	7				
MOL000173	Wogonin	MCL1	4	7				
MOL000173	Wogonin	MYC	4	7				
MOL000173	Wogonin	PTGS2	4	7				
MOL000173	Wogonin	TMPRSS11D	4	7				
MOL000211	Mairin	AKT1	9	10	11			
MOL000211	Mairin	CASP3	9	10	11			
MOL000211	Mairin	CYCS	9	10	11			
MOL000211	Mairin	EGFR	9	10	11			
MOL000211	Mairin	MAPK1	9	10	11			
MOL000211	Mairin	MAPK3	9	10	11			
MOL000211	Mairin	NOS3	9	10	11			
MOL000211	Mairin	TOP1	9	10	11			
MOL000211	Mairin	TOP2A	9	10	11			
MOL000354	Isorhamnetin	AKT1	10					
MOL000354	Isorhamnetin	CYP1B1	10					
MOL000354	Isorhamnetin	HMOX1	10					
MOL000354	Isorhamnetin	MAPK8	10					
MOL000354	Isorhamnetin	NOS2	10					
MOL000354	Isorhamnetin	NOS3	10					
MOL000358	Beta-sitosterol	PTGS2	1	3	7	9	11	
MOL000358	Beta-sitosterol	KCNH2	1	3	7	9	11	
MOL000358	Beta-sitosterol	SCN5A	1	3	7	9	11	
MOL000358	Beta-sitosterol	BAX	1	3	7	9	11	
MOL000358	Beta-sitosterol	JUN	1	3	7	9	11	
MOL000358	Beta-sitosterol	CASP3	1	3	7	9	11	
MOL000358	Beta-sitosterol	TGFB1	1	3	7	9	11	
MOL000359	Sitosterol	APOE	1	4	7	8	9	10
MOL000359	Sitosterol	CASP3	1	4	7	8	9	10
MOL000359	Sitosterol	ICAM1	1	4	7	8	9	10
MOL000392	Formononetin	CASP3	10					
MOL000392	Formononetin	CYP1B1	10					
MOL000392	Formononetin	UGT1A1	10					

MOL000392	Formononetin	UGT1A8	10		
MOL000417	Calycosin	MAPK1	10		
MOL000417	Calycosin	MAPK3	10		
MOL000417	Calycosin	UGT1A1	10		
MOL000417	Calycosin	UGT1A8	10		
MOL000422	Kaempferol	AKT1	3	9	10
MOL000422	Kaempferol	CASP3	3	9	10
MOL000422	Kaempferol	CCL2	3	9	10
MOL000422	Kaempferol	CSF2	3	9	10
MOL000422	Kaempferol	CYP1A2	3	9	10
MOL000422	Kaempferol	CYP1B1	3	9	10
MOL000422	Kaempferol	CYP3A4	3	9	10
MOL000422	Kaempferol	ESR1	3	9	10
MOL000422	Kaempferol	HMOX1	3	9	10
MOL000422	Kaempferol	IL2	3	9	10
MOL000422	Kaempferol	JUN	3	9	10
MOL000422	Kaempferol	MAPK1	3	9	10
MOL000422	Kaempferol	MAPK3	3	9	10
MOL000422	Kaempferol	NFKBIA	3	9	10
MOL000422	Kaempferol	NOS2	3	9	10
MOL000422	Kaempferol	SRC	3	9	10
MOL000422	Kaempferol	STAT1	3	9	10
MOL000422	Kaempferol	STAT3	3	9	10
MOL000422	Kaempferol	TP53	3	9	10
MOL000422	Kaempferol	UGT1A1	3	9	10
MOL000422	Kaempferol	UGT1A8	3	9	10
MOL000449	Stigmasterol	NR3C2	1	3	11
MOL000449	Stigmasterol	PTGS2	1	3	11
MOL000449	Stigmasterol	SCN5A	1	3	11
MOL000471	Aloe-emodin	CASP3	5		
MOL000471	Aloe-emodin	TP53	5		
MOL000492	(+)-Catechin	ESR1	9	11	
MOL000492	(+)-Catechin	PTGS2	9	11	
MOL000497	Licochalcone a	CXCL8	10		
MOL000500	Vestitol	CYP3A4	10		
MOL000627	Stepholidine	KCNH2	11		

MOL000627	Stepholidine	SCN5A	11
MOL000627	Stepholidine	PTGS2	11
MOL000627	Stepholidine	CA2	11
MOL000785	Palmatine	CYP2D6	2
MOL000785	Palmatine	CYP3A4	2
MOL000785	Palmatine	TOP1	2
MOL000787	Fumarine	KCNH2	11
MOL000787	Fumarine	SCN5A	11
MOL000787	Fumarine	PTGS2	11
MOL000787	Fumarine	CACNA1S	11
MOL000787	Fumarine	TOP2A	11
MOL001454	Berberine	ADIPOQ	11
MOL001454	Berberine	AKT1	11
MOL001454	Berberine	BECN1	11
MOL001454	Berberine	CASP3	11
MOL001454	Berberine	CTSB	11
MOL001454	Berberine	CYP2D6	11
MOL001454	Berberine	DPP4	11
MOL001454	Berberine	EGFR	11
MOL001454	Berberine	GCG	11
MOL001454	Berberine	GSK3B	11
MOL001454	Berberine	HMOX1	11
MOL001454	Berberine	IL17A	11
MOL001454	Berberine	ITGAM	11
MOL001454	Berberine	JUN	11
MOL001454	Berberine	MAPK1	11
MOL001454	Berberine	MAPK14	11
MOL001454	Berberine	NOS2	11
MOL001454	Berberine	NOS3	11
MOL001454	Berberine	PTGS2	11
MOL001454	Berberine	SRC	11
MOL001454	Berberine	STAT3	11
MOL001454	Berberine	TLR4	11
MOL001454	Berberine	TNF	11
MOL001454	Berberine	TOP1	11
MOL001454	Berberine	TP53	11

MOL001454	Berberine	NOS2	11		
MOL001454	Berberine	KCNH2	11		
MOL001454	Berberine	ESR1	11		
MOL001454	Berberine	SCN5A	11		
MOL001454	Berberine	PTGS2	11		
MOL001454	Berberine	NOS3	11		
MOL001458	Coptisine	XPO1	2	4	
MOL001506	Supraene	CCL2	4		
MOL001506	Supraene	CCL3	4		
MOL001506	Supraene	CCL4	4		
MOL001506	Supraene	CCL5	4		
MOL001506	Supraene	CXCL8	4		
MOL001506	Supraene	IL1RN	4		
MOL001506	Supraene	SEC14L2	4		
MOL001522	(S)-coclaurine	SCN5A	11		
MOL001522	(S)-coclaurine	PTGS2	11		
MOL001522	(S)-coclaurine	CA2	11		
MOL001689	Acacetin	CYP1A2	4	5	6
MOL001689	Acacetin	CYP1B1	4	5	6
MOL001689	Acacetin	IL13	4	5	6
MOL001689	Acacetin	IL5	4	5	6
MOL001689	Acacetin	JUN	4	5	6
MOL001689	Acacetin	STAT1	4	5	6
MOL001689	Acacetin	VEGFA	4	5	6
MOL001924	Paeoniflorin	CXCL8	9		
MOL001924	Paeoniflorin	TLR4	9		
MOL001941	Ammidin	CASP1	7		
MOL001941	Ammidin	CASP1	7		
MOL001941	Ammidin	CYP1B1	7		
MOL001942	Isoimperatorin	CYP1A2	7		
MOL001942	Isoimperatorin	CYP1B1	7		
MOL001942	Isoimperatorin	CYP2D6	7		
MOL002707	Phytofluene	EFNA5	3		
MOL002714	Baicalein	AKT1	4		
MOL002714	Baicalein	CDK4	4		
MOL002714	Baicalein	CFTR	4		

MOL002714	Baicalein	CYP1A2	4	
MOL002714	Baicalein	CYP1B1	4	
MOL002714	Baicalein	CYP3A4	4	
MOL002714	Baicalein	GSK3B	4	
MOL002714	Baicalein	HSD11B2	4	
MOL002714	Baicalein	IL6	4	
MOL002714	Baicalein	INS	4	
MOL002714	Baicalein	MAPK1	4	
MOL002714	Baicalein	MAPK3	4	
MOL002714	Baicalein	MAPK8	4	
MOL002714	Baicalein	MCL1	4	
MOL002714	Baicalein	NFE2L2	4	
MOL002714	Baicalein	NOS2	4	
MOL002714	Baicalein	NOS3	4	
MOL002714	Baicalein	NOTCH1	4	
MOL002714	Baicalein	PKM	4	
MOL002714	Baicalein	PTGS2	4	
MOL002714	Baicalein	SERPINE1	4	
MOL002714	Baicalein	TP53	4	
MOL002714	Baicalein	VEGFA	4	
MOL002773	Beta-carotene	CYP3A4	3	11
MOL002773	Beta-carotene	SCARB1	3	11
MOL002773	Beta-carotene	TMPRSS11D	3	11
MOL002773	Beta-carotene	AKT1	3	11
MOL002773	Beta-carotene	VEGFA	3	11
MOL002773	Beta-carotene	JUN	3	11
MOL002773	Beta-carotene	CASP3	3	11
MOL002773	Beta-carotene	PTGS2	3	11
MOL002773	Beta-carotene	HMOX1	3	11
MOL002773	Beta-carotene	CYP3A4	3	11
MOL002773	Beta-carotene	CYP1A2	3	11
MOL002773	Beta-carotene	ALB	3	11
MOL002773	Beta-carotene	CAV1	3	11
MOL002773	Beta-carotene	MYC	3	11
MOL002773	Beta-carotene	F3	3	11
MOL002881	Diosmetin	CYP1B1	5	

MOL002881	Diosmetin	CYP2C8	5		
MOL002881	Diosmetin	PKM	5		
MOL002928	Oroxylin a	BDNF	4		
MOL002928	Oroxylin a	IL6	4		
MOL002928	Oroxylin a	MAPK1	4		
MOL002928	Oroxylin a	MAPK3	4		
MOL002928	Oroxylin a	NOS2	4		
MOL002928	Oroxylin a	NOS3	4		
MOL002928	Oroxylin a	SIRT3	4		
MOL002932	Panicolin	CASP3	4		
MOL003036	Zinc03978781	CXCL8	3		
MOL003036	Zinc03978781	IL10	3		
MOL003036	Zinc03978781	TNF	3		
MOL003044	Chryseriol	CYP1B1	3		
MOL004328	Naringenin	ADIPOQ	5	8	10
MOL004328	Naringenin	BDNF	5	8	10
MOL004328	Naringenin	CCL2	5	8	10
MOL004328	Naringenin	CYP1A2	5	8	10
MOL004328	Naringenin	CYP1B1	5	8	10
MOL004328	Naringenin	HMOX1	5	8	10
MOL004328	Naringenin	KCNH2	5	8	10
MOL004328	Naringenin	MAPK14	5	8	10
MOL004328	Naringenin	TLR2	5	8	10
MOL004328	Naringenin	TNF	5	8	10
MOL004350	Ruvoside_qt	NR3C2	11		
MOL004580	Cis-Dihydroquercetin	NQO1	6		
MOL004841	Licochalcone B	CASP3	10		
MOL004908	Glabridin	ICAM1	10		
MOL004908	Glabridin	NOS2	10		
MOL004908	Glabridin	NOS3	10		
MOL004908	Glabridin	TTR	10		
MOL004908	Glabridin	TYR	10		
MOL004908	Glabridin	UGT1A1	10		
MOL004908	Glabridin	UGT1A8	10		
MOL005190	Eriodictyol	HMOX1	5		
MOL005190	Eriodictyol	TBK1	5		

MOL005573	Genkwanin	CYP1A2	5
MOL005573	Genkwanin	CYP1B1	5
MOL005573	Genkwanin	DUSP1	5
MOL005828	Nobiletin	CFTR	1
MOL005828	Nobiletin	CYP1A2	1
MOL005828	Nobiletin	MME	1
MOL005828	Nobiletin	MYC	1
MOL005828	Nobiletin	SNCA	1
MOL005828	Nobiletin	TMPRSS11D	1
MOL005828	Nobiletin	VEGFA	1
MOL007213	Nuciferin	SCN5A	11
MOL007213	Nuciferin	PTGS2	11
MOL009042	Helenalin	ATG12	1
MOL009042	Helenalin	MAPK8	1
MOL009042	Helenalin	NFKBIA	1
MOL009042	Helenalin	TBK1	1
MOL012921	Stepharine	SCN5A	11
MOL012921	Stepharine	PTGS2	11
MOL012946	Zizyphus saponin I_qt	NR3C2	11
MOL012976	Coumestrol	PPARG	11
MOL012976	Coumestrol	PTGS2	11
MOL012976	Coumestrol	CYP3A4	11
MOL012986	Jujubasaponin V_qt	NR3C2	11
MOL012992	Mauritine D	SCN5A	11
MOL012992	Mauritine D	PTGS2	11
MOL013352	Obacunone	CASP3	2
MOL013352	Obacunone	PTGS2	2

Table A5 List of gene targets in PPI network with average number of neighbors greater than 43.864.

Target name	Degree	Betweenness Centrality	Closeness Centrality
TNF	114	0.059158571	0.641176471
STAT3	114	0.055442145	0.637426901
IL6	112	0.050465153	0.622857143
TP53	100	0.101162876	0.612359551
AKT1	96	0.046266916	0.608938547
JUN	94	0.038398374	0.589189189
IL1B	90	0.019249483	0.573684211
ALB	86	0.135958006	0.608938547
VEGFA	84	0.028669235	0.589189189
TLR4	76	0.023630211	0.564766839
IL10	76	0.011536368	0.547738693
EGFR	74	0.014952245	0.570680628
CASP3	74	0.022575619	0.56185567
MAPK3	72	0.018364837	0.573684211
CXCL8	72	0.011030353	0.547738693
INS	68	0.024729408	0.564766839
IL2	68	0.006886173	0.536945813
SRC	66	0.045791764	0.545
STAT1	66	0.011422844	0.5215311
MYC	64	0.027884547	0.547738693
CCL2	62	0.005033402	0.5215311
IFNG	58	0.003172539	0.514150943
NFKBIA	56	0.013971527	0.524038462
CSF2	56	0.002292287	0.50462963
CCL4	54	0.011005734	0.5

Table A6 List of top 10 site of expression

Site of expression	No. of genes	Percentage of genes	*p*-value
Endometrium	44	37.28813559	2.06331E-22
Thyroid	35	29.66101695	1.34894E-18
Uterine cervix	33	27.96610169	1.42796E-16
Vulva	31	26.27118644	1.07776E-15
Oesophagus	31	26.27118644	1.42065E-14
Urinary bladder	85	72.03389831	1.43624E-13
Tonsils	86	72.88135593	2.59857E-13
Skin	86	72.88135593	3.05769E-13
Nasopharynx	82	69.49152542	2.32794E-12
Lung	100	84.74576271	3.08376E-12

Table A7 List of top 10 biological pathways.

Biological pathway	No. of genes	Percentage of genes	*p*-value
TNF receptor signaling pathway	35	33.65384615	2.1966E-21
AP-1 transcription factor network	47	45.19230769	4.8682E-21
Integrin-linked kinase signaling	47	45.19230769	4.39422E-20
IL1-mediated signaling events	30	28.84615385	2.1809E-19
Integrin family cell surface interactions	64	61.53846154	1.5283E-18
IFN-gamma pathway	62	59.61538462	2.00333E-18
ALK1 signaling events	33	31.73076923	2.42927E-18
CDC42 signaling events	48	46.15384615	2.62954E-18
Endothelins	62	59.61538462	3.13309E-18
ALK1 pathway	33	31.73076923	3.24213E-18

Table A8 List of top 20 GO terms of biological process enrichment analysis.

GO term	Gene number	P-value	Rich Factor
response to lipopolysaccharide	38	-42.98033797	0.111
response to molecule of bacterial origin	38	-42.01363029	0.105
response to bacterium	45	-39.32242325	0.06
positive regulation of cell death	41	-37.27395981	0.066
cellular response to biotic stimulus	31	-36.57607225	0.127
positive regulation of programmed cell death	38	-34.88613135	0.068
response to drug	34	-34.25741043	0.085
cellular response to lipopolysaccharide	28	-33.7325553	0.135
cellular response to molecule of bacterial origin	28	-33.01957066	0.127
positive regulation of apoptotic process	36	-32.56161147	0.067
positive regulation of cell migration	36	-32.08121652	0.065
positive regulation of cytokine production	34	-31.92681577	0.073
response to inorganic substance	36	-31.58956641	0.063
apoptotic signaling pathway	36	-31.37638056	0.062
positive regulation of cell motility	36	-31.37638056	0.062
positive regulation of cellular component movement	36	-31.06247353	0.061
positive regulation of locomotion	36	-31.01082326	0.06
cellular response to lipid	35	-30.21770158	0.061
regulation of neuron death	29	-29.87420204	0.091
neuron death	30	-29.75465373	0.083

Table A9 List of top 20 GO terms of cellular component enrichment analysis.

GO term	Gene number	P-value	Rich Factor
membrane raft	19	2.58E-16	0.056
membrane microdomain	19	2.58E-16	0.056
plasma membrane raft	11	2.60E-12	0.094
caveola	10	2.72E-12	0.118
focal adhesion	9	7.29E-05	0.021
cell-substrate junction	9	8.26E-05	0.021
late endosome	5	0.006787944	0.018
vesicle lumen	16	6.06E-13	0.049
cytoplasmic vesicle lumen	15	7.71E-12	0.046
secretory granule lumen	14	8.83E-11	0.043
platelet alpha granule	6	2.37E-06	0.066
platelet alpha granule lumen	5	9.31E-06	0.075
perinuclear region of cytoplasm	19	2.58E-10	0.026
endoplasmic reticulum lumen	13	7.55E-10	0.042
endocytic vesicle	13	1.78E-09	0.039
endocytic vesicle membrane	9	1.30E-07	0.047
early endosome	10	7.62E-06	0.025
clathrin-coated endocytic vesicle membrane	4	0.000241907	0.056
clathrin-coated endocytic vesicle	4	0.000591185	0.044
clathrin-coated vesicle membrane	4	0.00146174	0.034

Table A10 List of top 20 GO terms of molecular function enrichment analysis.

GO term	Gene number	P-value	Rich Factor
cytokine receptor binding	26	4.64784E-28	0.096
signaling receptor activator activity	31	9.5637E-28	0.063
receptor ligand activity	30	1.24146E-26	0.062
signaling receptor regulator activity	31	1.59675E-26	0.057
cytokine activity	23	4.55467E-25	0.098
growth factor receptor binding	12	8.37163E-13	0.085
growth factor activity	10	1.7162E-09	0.062
protein homodimerization activity	23	9.06509E-15	0.034
heme binding	12	7.05167E-13	0.086
tetrapyrrole binding	12	1.62117E-12	0.081
monooxygenase activity	9	5.27401E-10	0.087
oxidoreductase activity, acting on paired donors, with incorporation or reduction of molecular oxygen	10	5.01786E-09	0.055
oxidoreductase activity, acting on paired donors, with incorporation or reduction of molecular oxygen, reduced flavin or flavoprotein as one donor, and incorporation of one atom of oxygen	6	1.62701E-08	0.15
estrogen 16-alpha-hydroxylase activity	4	2.02418E-08	0.5
oxidoreductase activity	16	9.14339E-08	0.022
oxidoreductase activity, acting on CH or CH2 groups, quinone or similar compound as acceptor	3	2.86024E-07	0.75
caffeine oxidase activity	3	2.86024E-07	0.75
steroid hydroxylase activity	5	5.33102E-07	0.132
aromatase activity	4	3.46193E-06	0.16
iron ion binding	7	3.55396E-06	0.046

Table A11 List of top 20 KEGG signaling pathways of enrichment analysis.

KEGG pathway	Gene number	*p*-value	Rich Factor
AGE-RAGE signaling pathway in diabetic complications	25	-36.52127217	0.234
Chagas disease (American trypanosomiasis)	23	-33.17360542	0.225
IL-17 signaling pathway	22	-32.23213202	0.237
Influenza A	24	-29.16711449	0.139
Toll-like receptor signaling pathway	21	-29.16211391	0.202
Leishmania infection	18	-26.73066741	0.247
Leishmaniasis	18	-26.73066741	0.247
Tuberculosis	22	-25.50977173	0.123
Toxoplasmosis	19	-24.74948074	0.168
Pertussis	17	-24.45003798	0.224
Salmonella infection	17	-23.44721208	0.198
TNF signaling pathway	17	-21.63958387	0.157
NOD-like receptor signaling pathway	19	-21.2198583	0.112
T cell receptor signaling pathway	15	-18.58846765	0.146
Th17 cell differentiation	15	-18.32799797	0.14
Non-alcoholic fatty liver disease (NAFLD)	16	-16.724634	0.095
MAPK signaling pathway	18	-16.49380505	0.071
Osteoclast differentiation	14	-15.47011159	0.108
Th1 and Th2 cell differentiation	12	-14.3445395	0.13
Hepatitis C	25	-31.13290586	0.148

Table A12 Primer sequences of genes used for mouse RAW264.7 cells and human Calu-3 cells in quantitative real-time PCR analysis.

Mouse	Forward (5' to 3')	Reverse (5' to 3')
**NFκB2**	TGCTGATGGCACAGGACGAGAA	GTTGATGACGCCGAGGTACTGA
**c-Rel**	GAAGACTGCGACCTCAATGTGG	TCTTGTTCACACGGCAGATCCTT
**IL-1β**	TGCCACCTTTTGACAGTGATG	AAGGTCCACGGGAAAGACAC
**IL-6**	ACTTCACAAGTCGGAGGCTT	TGTGACTCCAGCTTATCTCTTGG
**TNF-α**	AGGCACTCCCCCAAAAGATG	CCACTTGGTGGTTTGTGAGTG
**CCL2**	GCTACAAGAGGATCACCAGCAG	GTCTGGACCCATTCCTTCTTGG
**NOS2**	GAGACAGGGAAGTCTGAAGCAC	CCAGCAGTAGTTGCTCCTCTTC
**β-Actin**	TAGGCGGACTGTTACTGAGC	TGCTCCAACCAACTGCTGTC

Human	Forward (5' to 3')	Reverse (5' to 3')
**TGF-β**	TACCTGAACCCGTGTTGCTCTC	GTTGCTGAGGTATCGCCAGGAA
**CXCL16**	CCTATGTGCTGTGCAAGAGGAG	CTGGGCAACATAGAGTCCGTCT
**CXCR6**	CAGTTCAGCAAGGTCTTTCTGCC	AGGTTCACCAGGAACACATCCG
**IL-17**	CGGACTGTGATGGTCAACCTGA	GCACTTTGCCTCCCAGATCACA
**IL-25**	AACCGCCACCCAGAGTCCTGT	ACAGGCAACGGGCGTGGTACA
**α-SMA**	CTATGCCTCTGGACGCACAACT	CAGATCCAGACGCATGATGGCA
**Col-1a**	GATTCCCTGGACCTAAAGGTGC	AGCCTCTCCATCTTTGCCAGCA
**Col-4a**	TGTTGACGGCTTACCTGGAGAC	GGTAGACCAACTCCAGGCTCTC
**Elastin**	GGTTGTGTCACCAGAAGCAGCT	CCGTAAGTAGGAATGCCTCCAAC
**Fn1**	CCGCTTGTCCTCCTTCTCGTTC	GGACACAACGATGCTTCCTGAG
**β-Actin**	TGAGCGCGGCTACAGCTT	TCCTTAATGTCACGCACGATTT
